# Myosin IIA-related Actomyosin Contractility Mediates Oxidative Stress-induced Neuronal Apoptosis

**DOI:** 10.3389/fnmol.2017.00075

**Published:** 2017-03-14

**Authors:** Yan Wang, Yingqiong Xu, Qian Liu, Yuanyuan Zhang, Zhen Gao, Mingzhu Yin, Nan Jiang, Guosheng Cao, Boyang Yu, Zhengyu Cao, Junping Kou

**Affiliations:** ^1^State Key Laboratory of Natural Products, Jiangsu Key Laboratory of TCM Evaluation and Translational Research, Department of Complex Prescription of TCM, China Pharmaceutical UniversityNanjing, China; ^2^Department of Neurology, Jinling Hospital, Nanjing University School of MedicineNanjing, China; ^3^Department of Medicine-Ather and Lipo, Baylor College of MedicineHouston, TX, USA; ^4^Department of Pathology, Yale School of MedicineNew Haven, CT, USA

**Keywords:** oxidative stress, neuronal apoptosis, myosin IIA, myosin IIB, actomyosin contractility, positive feedback loop

## Abstract

Oxidative stress-induced neuronal apoptosis plays an important role in the progression of central nervous system (CNS) diseases. In our study, when neuronal cells were exposed to hydrogen peroxide (H_2_O_2_), an exogenous oxidant, cell apoptosis was observed with typical morphological changes including membrane blebbing, neurite retraction and cell contraction. The actomyosin system is considered to be responsible for the morphological changes, but how exactly it regulates oxidative stress-induced neuronal apoptosis and the distinctive functions of different myosin II isoforms remain unclear. We demonstrate that myosin IIA was required for neuronal contraction, while myosin IIB was required for neuronal outgrowth in normal conditions. During H_2_O_2_-induced neuronal apoptosis, myosin IIA, rather than IIB, interacted with actin filaments to generate contractile forces that lead to morphological changes. Moreover, myosin IIA knockout using clustered regularly interspaced short palindromic repeats/CRISPR-associated protein-9 nuclease (CRISPR/Cas9) reduced H_2_O_2_-induced neuronal apoptosis and the associated morphological changes. We further demonstrate that caspase-3/Rho-associated kinase 1 (ROCK1) dependent phosphorylation of myosin light chain (MLC) was required for the formation of the myosin IIA-actin complex. Meanwhile, either inhibition of myosin II ATPase with blebbistatin or knockdown of myosin IIA with siRNA reversely attenuated caspase-3 activation, suggesting a positive feedback loop during oxidative stress-induced apoptosis. Based on our observation, myosin IIA-actin complex contributes to actomyosin contractility and is associated with the positive feedback loop of caspase-3/ROCK1/MLC pathway. This study unravels the biochemical and mechanistic mechanisms during oxidative stress-induced neuronal apoptosis and may be applicable for the development of therapies for CNS diseases.

## Introduction

Oxidative stress-induced neuronal apoptosis has been implicated in many central nervous system (CNS) diseases, including Alzheimer’s diseases, stroke and schizophrenia (Hayashi-Takagi et al., 2014; Volpe and Paneni, 2015; Kamat et al., 2016). One of the major pathological features of these neuronal disorders is the dysfunction of cytoskeleton, which influences synapse formation and maturation, vesicle/organelle trafficking, axon regeneration and spine morphology (Schafer et al., 2009; Gordon-Weeks and Fournier, 2014; Hayashi-Takagi et al., 2014). It has been demonstrated that the actomyosin system is responsible for the structural and morphological changes during the execution of apoptosis, such as dynamic cell contraction, membrane blebbing and chromatin condensation (Croft et al., 2005; Even-Ram et al., 2007; Ndozangue-Touriguine et al., 2008). The actomyosin system consists of actin filaments and myosin. Among the members of the myosin family, myosin II motor protein has been implicated in producing the forces responsible for cellular contraction (Landino and Ohi, 2016). Myosin II is a hexameric polypeptide, comprised of two myosin heavy chains (MHC) and two sets of myosin light chains (MLC). The N-terminal globular motor domain of MHC uses the energy of ATP hydrolysis to generate force and move along actin filaments, while C-terminal tail region interacts with other MHCs to form anti-parallel thick filaments (Bresnick, 1999; Hartman and Spudich, 2012). In the CNS, myosin II has been demonstrated to regulate neuronal morphogenesis (Ozkan et al., 2015), migration (Solecki et al., 2009), axon outgrowth (Pool et al., 2011), as well as growth cone motility (Medeiros et al., 2006). However, how exactly the actomyosin system regulates oxidative stress-induced neuronal apoptosis remains unclear.

There are two major isoforms of myosin II in neuronal cells, myosin IIA and IIB, which are encoded by *Myh9* and *Myh10*, respectively (Simons et al., 1991). Due to a high degree of homology in the amino acid sequences of their heavy chains, myosin IIA and IIB have overlapping cellular functions (Wylie and Chantler, 2001). However, they also display different catalytic activities, molecular interactions, tissue and intracellular distributions (Vicente-Manzanares et al., 2007; Zhou and Wang, 2008). Myosin IIB is the predominate isoform in the nervous system, driving neurite outgrowth processes (Rex et al., 2010; Yu et al., 2012), and modulating dendritic spines morphology and synaptic function (Ryu et al., 2006), while myosin IIA is required in maintaining tensile adhesion (Wylie and Chantler, 2001) and neurite retraction (Wylie and Chantler, 2003). In our previous studies, we have demonstrated that the neuroprotective mechanism of Ginsenoside Rg1 is associated with the inhibition of myosin IIA-actin interaction (Wang et al., 2016). However, little knowledge is available on the distinct roles of myosin II isoforms in oxidative stress-induced neuronal apoptosis. We are interested to investigate how myosin II isoforms regulate neuronal apoptosis.

Moreover, the actin-myosin interaction is highly regulated by the phosphorylation of the regulatory MLC, through MLC kinase (MLCK), MLC phosphatase and Rho-associated protein kinase (ROCK1 and ROCK2; Nilius et al., 2000; Totsukawa et al., 2000). ROCK is a serine/threonine protein kinase that directly phosphorylates MLC or indirectly increases MLC phosphorylation by inactivating myosin light chain phosphatase (MLCP). Activated Rho binds to ROCK and disrupts the negative regulatory sequence between the kinase domain and the autoinhibitory region, resulting in a truncated active form (Kimura et al., 1996; Eitaki et al., 2012). In addition, only ROCK1 can be cleaved by caspase-3, generating active kinase to induce apoptosis (Coleman et al., 2001). Inhibition of ROCK or caspase activity abrogates MLC phosphorylation and apoptotic membrane blebbing in astrocytes and TF-1 cells (Lai et al., 2003; Miñambres et al., 2006). We have previously illustrated the caspase-3/ROCK1/MLC feedback loop in hydrogen peroxide (H_2_O_2_)-induced PC12 cells injury (Shen et al., 2015a), but the correlation between myosin II and the positive feedback loop remains unknown.

Our study focuses on the distinctive contribution of myosin IIA and IIB in H_2_O_2_-induced neuronal apoptosis and the related mechanisms. We demonstrate that myosin IIA displays a distinctive role in the formation of functional contractile cytoskeleton that is required for apoptotic membrane blebbing and neurite retraction, whereas myosin IIB drives the neuronal outgrowth processes. The myosin IIA-actin interaction regulates caspase-3/ROCK1/MLC signal cascade by a feedback mechanism upon oxidative stress. These data indicate that myosin II isoforms function differently in cytoskeletal reorganization and only myosin IIA functions in positive feedback loop pathway in neuronal apoptosis. It provides new insights on the mechanisms of oxidative stress-induced neuronal apoptosis and paves the way for development of better treatments for neuronal diseases.

## Materials and Methods

### Material

L-glutamine, Neurobasal medium, B-27 supplement (50×, minus antioxidants), soybean trypsin inhibitor, Dulbecco’s modified Eagle’s medium (DMEM), Opti-modified Eagle’s medium^®^ (Opti-MEM^®^), Lipofectamine^®^ RNAiMAX, Lipofectamine^®^ 3000, Alexa Fluor^®^ 488 donkey anti-rabbit antibody and Alexa Fluor^®^ 568 phalloidin were obtained from Thermo Fisher Scientific (San Jose, CA, USA). Fetal bovine serum was from ScienCell (San Diego, CA, USA). Penicillin, streptomycin and 3-(4,5-dimethylthiazol-2-yl)-2,5-diphenyl tetrazolium bromide (MTT) were purchased from Amresco (Solon, OH, USA). DNase, cytosine arabinoside, poly-L-lysine, N-acetyl-L-cysteine (NAC), blebbistatin, cytochalasin D, ML-7 and H_2_O_2_ and were from Sigma-Aldrich (St. Louis, MO, USA). Y27632, z-VAD-fmk and z-DEVD-fmk were from Selleck Chemicals (Houston, TX, USA). Anti-MLC, anti-P-MLC (Ser19), anti-caspase-3, anti-microtubule-associated protein 2 (MAP2) antibodies were from Cell Signaling Technology (Danvers, MA, USA). Anti-myosin IIA and anti-actin antibodies were from Abcam (Cambridge, UK). Anti-ROCK1 and anti-ROCK2 antibodies, Protein A/G PLUS-Agarose, normal rabbit IgG and normal mouse IgG were from Santa Cruz Biotechnology (Dallas, TX, USA). Anti-GAPDH antibody was purchased from KangCheng (Shanghai, China). HRP-conjugated secondary antibodies were from Boster (Wuhan, China). DAPI was from Beyotime Biotech (Haimen, China). Protease inhibitor cocktail, RIPA buffer and ECL were purchased from Vazyme Biotech (Nanjing, China). Dimethyl sulfoxide (DMSO) was obtained from SunShineBio (Nanjing, China).

### Cell Culture

Highly differentiated PC12 (rat adrenal pheochromocytoma) cells were purchased from Shanghai Institute of Cell Biology, Chinese Academy of Sciences. According to the protocol from Shanghai Institute of Cell Biology, PC12 cells were differentiated in differentiation medium (DMEM with 100 ng/ml nerve growth factor) for 5–7 days to induce neurite formation and differentiation. The media was changed with new differentiation medium every 2–3 days. The differentiated PC12 cells were cultured in DMEM, supplemented with 10% fetal bovine serum, 100 I.U./mL penicillin and 100 μg/mL streptomycin, in a humidified cell culture incubator in 5% CO_2_ atmosphere at 37°C.

### Primary Cortical Neurons Cultures

Primary cultures of cortical neurons were obtained from Sprague-Dawley rats on embryonic day 16–17 and processed as described (Cao et al., 2015). The pregnant Sprague-Dawley rats were provided by the Reference Animal Research Centre of Yangzhou University (Yangzhou, China; certificate no SCXK 2014-0004). The pregnant rats were euthanized by CO_2_ asphyxiation, and embryos were removed under sterile conditions. Neocortices were collected, removed of their meninges, minced by trituration with a Pasteur pipette, and digested in trypsin at 37°C for 25 min. The cells were further dissociated by two successive trituration and sedimentation steps in isolation buffer containing soybean trypsin inhibitor and DNase. The cells were then centrifuged and resuspended in Eagle’s minimal essential medium with Earle’s salt (MEM), supplemented with 2 mM L-glutamine, 10% fetal bovine serum, 100 I.U./mL penicillin and 100 μg/mL streptomycin, pH 7.4. The dissociated cells were plated onto poly-L-lysine-coated 96-well culture plates or 6-well culture plates at a density of 1 × 10^6^ cells/mL. For the immune-fluorescent experiments, cells were plated onto poly-L-lysine-coated 35-mm confocal dishes (Glass Bottom Dish) at a density of 2 × 10^5^ cells/mL. Cells were cultured at 37°C in a 5% CO_2_ and 95% humidity atmosphere. Cells were treated with Cytosine arabinoside (10 μM) after 24–48 h post plating to prevent proliferation of non-neuronal cells. The culture medium was changed every 3 days using Neurobasal medium supplemented with B-27 supplement (minus antioxidants), 2 mM L-glutamine, 100 I.U./mL penicillin and 100 μg/mL streptomycin, pH 7.4. The cultures were used for experiments between 7 and 9 DIV (days *in vitro*). This study was carried out in accordance with the National Institutes of Health Guide for the care and use of laboratory animals. Animal experiments and surgical procedures were approved by the Animal Ethics Committee of the School of Chinese Materia Medica, China Pharmaceutical University.

### Cell Treatment

Cells were treated with NAC (500 μM), blebbistatin (1 μM), cytochalasin D (1 μM), ML-7 (5 μM), Y27632 (10 μM), z-VAD-fmk (10 μM) or z-DEVD-fmk (10 μM) for 1 h, followed by co-incubating with 100 μM H_2_O_2_ for another 12 h. All compounds were dissolved in DMSO and finally diluted in the serum free medium with DMSO at the final concentration of 0.1%. DMSO was added in the control groups.

### siRNA Transfection

The siRNA oligonucleotides used to knockdown myosin IIA or IIB were purchased from Biomics Biotech (Nantong, China). The antisense sequence for myosin IIA (*Myh9*) is 5′-GAGACAAUGGAGGCCAUGA-3′. The antisense sequence for myosin IIB (*Myh10*) is 5′-CUAUUCAGGACUCAUCUAU-3′. PC12 cells growing on 6-well plates at 50%–60% confluency were transfected with 5 μg of siRNA and 5 μL of Lipofectamine^®^ RNAiMAX in the Opti-MEM^®^ medium. The cells were incubated with the medium containing siRNA for 6 h. The transfection medium was then replaced with complete medium without antibiotics. Cells were processed for analysis 48–72 h after transfection.

### Generation of Myh9 Knockout Cells Using CRISPR/Cas9 System

MYH9 clustered regularly interspaced short palindromic repeats/CRISPR-associated protein-9 nuclease (CRISPR/Cas9) knockout plasmid (Santa Cruz Biotechnology, Dallas, TX, USA) consists of a pool of three plasmids, encoding three guide RNAs (gRNAs) to target different regions of Myh9 gene for maximum knockout efficiency. The gRNAs sequences targeted by Myh9 CRISPR plasmids are 5′-TGGTTCAAAGCCATTCTTGG-3′, 5′-ACCAGCCAGCCTTAAGGAGG-3′ and 5′-TATCTACTCAGAGGAGATCG-3′. Control CRISPR/Cas9 Plasmid does not recognize any DNA sequence used as a negative control (NC). Each plasmid encodes Cas9 nuclease and green fluorescent protein (GFP). PC12 cells were seeded in 6-well plates (at a density of 1 × 10^6^ cells/well) the day before transfection. Cells were transfected with 2 μg of the plasmid using lipofectamine 3000^®^. After incubation of 72 h, GFP-positive cells were sorted and grown as single cell in 96-well plates using the BD FACSAria III cell sorter (BD Biosciences, San Jose, CA, USA). Colonies derived from single cells were expanded in media with 20% FBS. After 3 weeks, Myh9 knockout cells were screened by Western blotting using myosin IIA-specific antibodies.

### Cell Viability

Cell viability was determined by the MTT assay. Cells were seeded in 96-well plates and cultured at 37°C in an atmosphere of 5% CO_2_ and 95% relative humidity. Following treatment, the medium was aspirated, and 100 μL of culture medium containing 5 mg/mL of MTT dye was added to each well. Cells were incubated for 4 h. The reaction mixture was carefully taken out and 150 μL of DMSO was added to each well. The 96-well plates were shaken for 10 min and the absorbance was then recorded with dual waves at 570 and 650 nm by a microplate reader (Epoch, BioTek, Winooski, VT, USA). The cell viability was expressed as percentage of vehicle-treated controls.

### Measurement of Caspase-3 Activity

Caspase-3 activity was evaluated according to the manual of the Caspase-3 Activity Assay kit (Beyotime Shanghai, China). Briefly, the whole cell lysates (WCL) were added into 96-well plates and incubated with 2 mM of caspase-3 substrate (Ac-DEVD-pNA) at 37°C for 4 h. The absorbance was measured at 405 nm using a microplate reader. The protein levels of the samples were measured by the Bradford protein assay (Beyotime, Shanghai, China). Relative caspase-3 activity was expressed as percentage of vehicle-treated controls.

### Membrane Blebbing, Neurite Length and Cell Body Area of PC12 Cells

PC12 cells were seeded in 6-well plates at a density of 1 × 10^5^ cells/mL, treated as mentioned above, and observed with a phase contrast microscope (Olympus Corporation, Tokyo, Japan). PC12 cells were considered blebbing when blebs extended and cell bodies contracted. The percent of blebbing cells was determined by counting total and blebbing cells under blinded conditions. Neurite length was measured from the tip of the neurite until the soma of the cell using the ImageJ software (National Institutes of Health, Bethesda, MD, USA). Cell body area was measured using the ImageJ software. Neurite length and cell body area were presented as percentage of controls.

### Transmission Electron Microscopy (TEM)

Cell suspensions were centrifuged, resuspended in fixative in a Eppendorf tube, spun down again to form a pellet and stored overnight at 4°C. Cells were then postfixed in 1% osmium tetroxide in 0.10 M phosphate buffer, dehydrated in a graded series of ethanol (30, 50, 70, 80, 90 and 100%), infiltrated, and embedded in an “Epon-Araldite” mixture. Seventy-five nanometer sections were stained with uranyl acetate and bismuth subnitrite, and then examined in a transmission electron microscope (JEM-1010, JEOL Ltd, Tokyo, Japan) at 80 Kv accelerating voltage.

### Immunofluorescence

PC12 cells and neurons were washed with cold PBS, fixed in 4% paraformaldehyde, permeabilized with 0.1% Triton X-100 in PBS for 30 min at room temperature, blocked with 5% BSA and incubated with rabbit anti-myosin IIA antibody at 1:200 dilution or rabbit anti-MAP2 antibody at 1:200 dilution overnight at 4°C, followed by 1 h incubation with Alexa Fluor^®^ 488 donkey anti-rabbit antibody at 1:200 dilution at room temperature. Filamentous actin structures were stained with 1:200 dilution of Alexa Fluor^®^ 568 Phalloidin. Nuclei were visualized with DAPI. Fluorescent images were taken through laser excitation lines 405, 488, 568 nm and Differential Interference Contrast (DIC) with confocal laser scanning microscope (LSM700, Zeiss, Germany). Cells were imaged with the same optical slice thickness in every channel using the Objective Plan-Apochromat 63×/1.40 Oil DIC M27. The resolution of images is 1024 × 1024 and the pinhole is set to one Airy unit. Images were obtained using the ZEN imaging software (Zeiss, Germany). Quantitative co-localization of myosin IIA or IIB with actin filaments was performed using the ImageJ software which can provide the Manders’ coefficients for the overlap of the images. Its values range between 0 and 1.0. A value of 1 represents strong positive correlation and 0 indicates that there is no discernable correlation. Neurite length was quantified from neuronal cultures immunostained with MAP2 antibody. Neurite length was measured from the tip of the neurite until the soma of the neuron using the ImageJ software and was presented as percentage of control. All micrographs include a bar to indicate the scale.

### Western Blot

Cells were washed twice with PBS, lysed in RIPA buffer containing complete protease inhibitor cocktail and centrifuged at 12,000 rpm for 10 min at 4°C. Protein concentrations were determined according to BCA protein assay. Equal amounts of proteins (30 μg) were electrophoresed in 10% SDS-PAGE gels, transferred onto polyvinylidene fluoride (PVDF) membranes (Millipore Corporation, Billerica, MA, USA). The targeting proteins were probed with antibodies against myosin IIA (1:1000), myosin IIB (1:1000), ROCK1 (1:300), ROCK2 (1:300), MLC (1:1000), P-MLC (Ser19, 1:1000), caspase-3 (1:1000), GAPDH (1:10,000) and appropriate HRP-conjugated secondary antibodies (1:10,000), followed by visualization with ECL and photographed using the Bio-Rad Gel Imaging System (Bio-Rad, Hercules, CA, USA). Data analysis was done using ImageJ software measuring integrated density of bands after background subtraction.

### Immunoprecipitation

The Protein A/G PLUS-Agarose was washed with RIPA buffer three times before mixing with antibody. Prior to immunoprecipitation, 2 μg of purified antibodies against myosin IIA, actin filaments or normal IgG were mixed with 30 μL of agarose solution, and incubated at 4°C overnight on a rocker table. The prepared antibody-agarose complex were added to 1 mL of WCL (1 mg/mL) and incubated with rocking for 4 h at 4°C. The agarose-containing lysates were centrifuged and washed three times with RIPA buffer. Agarose-bound proteins were released by adding 2× SDS gel loading buffer and boiling for 5 min. An equal volume of each sample was fractionated by 10% SDS-PAGE and blotted using specific antibodies.

### Statistical Analysis

Data are presented as the means ± SD of three independent experiments. Statistical analysis of the data was performed with Student’s *t* test for two group comparison or one-way analysis of variance (ANOVA) followed by Dunnett’s *post hoc* test for multiple comparisons using GraphPad Prism 6.0 (GraphPad Software, La Jolla, CA, USA). *P* < 0.05 was considered statistically significant.

## Results

### H_2_O_2_ Induces Apoptosis and Membrane Blebbing in Neuronal Cells

**Figure 1:**
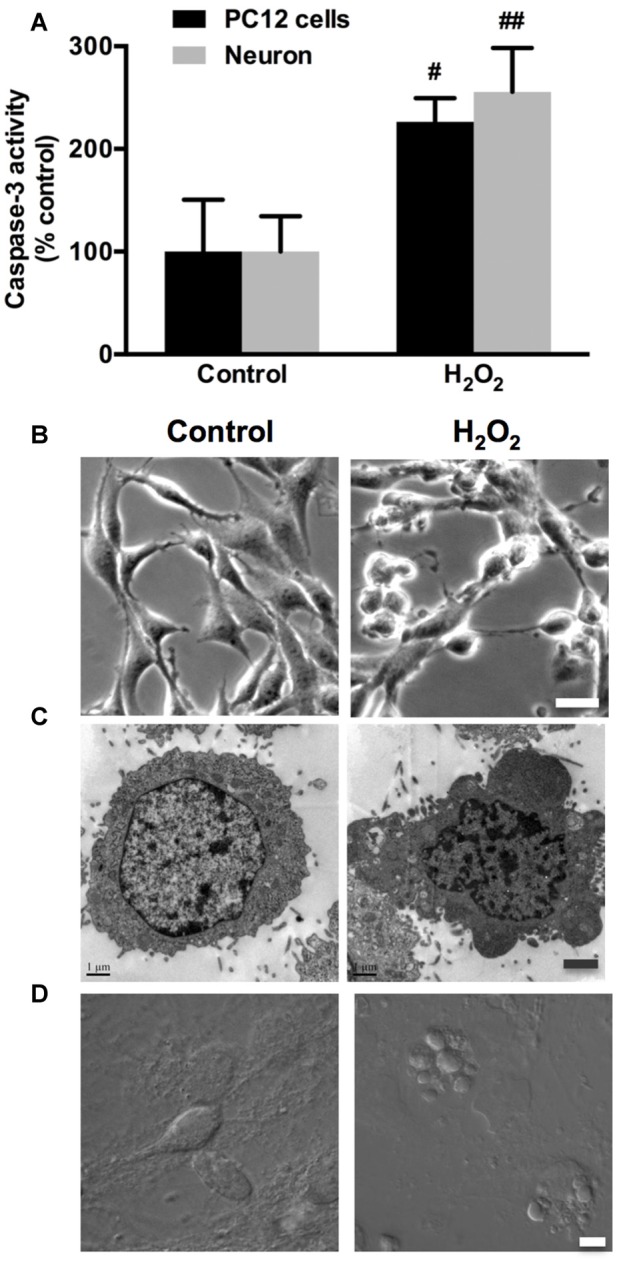
**Hydrogen peroxide (H_2_O_2_) induces apoptosis and membrane blebbing in PC12 cells and neurons. (A)** PC12 cells and neurons were exposed to 100 μM H_2_O_2_ for 12 h. Caspase-3 activity was evaluated in PC12 cells and neurons. **(B)** PC12 cells morphology using phase-contrast microscopy. Bar, 10 μm. **(C)** Transmission electron microscopy (TEM) of PC12 cells. Bar, 1 μm. **(D)** Neurons were imaged through differential interference contrast (DIC) using confocal laser scanning microscope (LSM). Bar, 5 μm. Results were expressed as mean ± SD from three independent experiments (^#^*P* < 0.05 vs. control, ^##^*P* < 0.01 vs. control).

In the present study, we used highly differentiated PC12 cells and primary cultured rat cortical neurons stimulated by H_2_O_2_ as the *in vitro* neuronal oxidative stress models. Caspase-3 activity significantly increased following 100 μM H_2_O_2_ treatment for 12 h in both PC12 cells and neurons (Figure 1A). The phase-contrast micrographs showed that normal PC12 cells had typical elongated and spreading morphology with extended neurites (Figure 1B, Control). Following H_2_O_2_ treatment, PC12 cells lost membrane extensions, detached from the culture support and shrunk to a rounded shape with obvious membrane blebbing (Figure 1B, H_2_O_2_). Transmission Electron Microscopy (TEM) was utilized to obtain fine-detailed photomicrographs of PC12 cells morphology. A typical apoptotic cell was observed with dramatic membrane blebs and chromatin condensation after H_2_O_2_ treatment (Figure 1C, H_2_O_2_). In contrast, control PC12 cells did not bleb and their nucleus remained intact (Figure 1C, Control). Consistently, similar results were also observed in neurons (Figure 1D).

### Myosin IIA-actin Interaction Mediates H_2_O_2_-induced Neuronal Apoptosis

**Figure 2:**
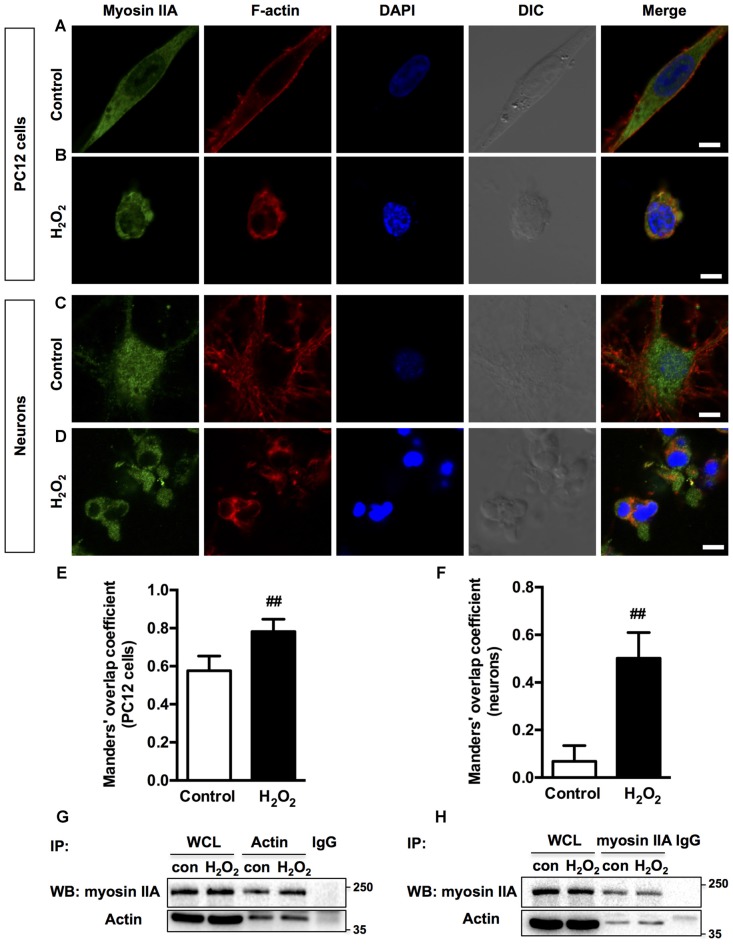
**Localization of myosin IIA and F-actin in PC12 cells or neurons upon H_2_O_2_ treatment.** PC12 cells untreated **(A)** or treated **(B)** with 100 μM H_2_O_2_ for 12 h were stained with myosin IIA (green), F-actin (red) and DAPI (blue). Neurons untreated **(C)** or treated **(D)** with 100 μM H_2_O_2_ for 12 h were stained with myosin IIA (green), F-actin (red) and DAPI (blue). Images were obtained by confocal microscopy. Bar, 5 μm. The co-localization of myosin IIA with F-actin in PC12 cells **(E)** or neurons **(F)** was evaluated on the basis of Manders’ overlap coefficients. Results were expressed as mean ± SD (^##^*P* < 0.01 vs. control). Experiments were performed three times independently. Protein interaction between myosin IIA and actin was determined by co-immunoprecipitation. Following treatment, cell lysates were immunoprecipitated with anti-actin antibody **(G)** or anti-non-muscle myosin IIA antibody **(H)**. Isotype-matched (IgG) served as negative control (NC). Each precipitated sample was detected for the presence of myosin IIA and actin by immunoblot analysis using specific antibodies. Whole cell lysates (WCL) prior to the immunoprecipitation served as input controls.

**Figure 3:**
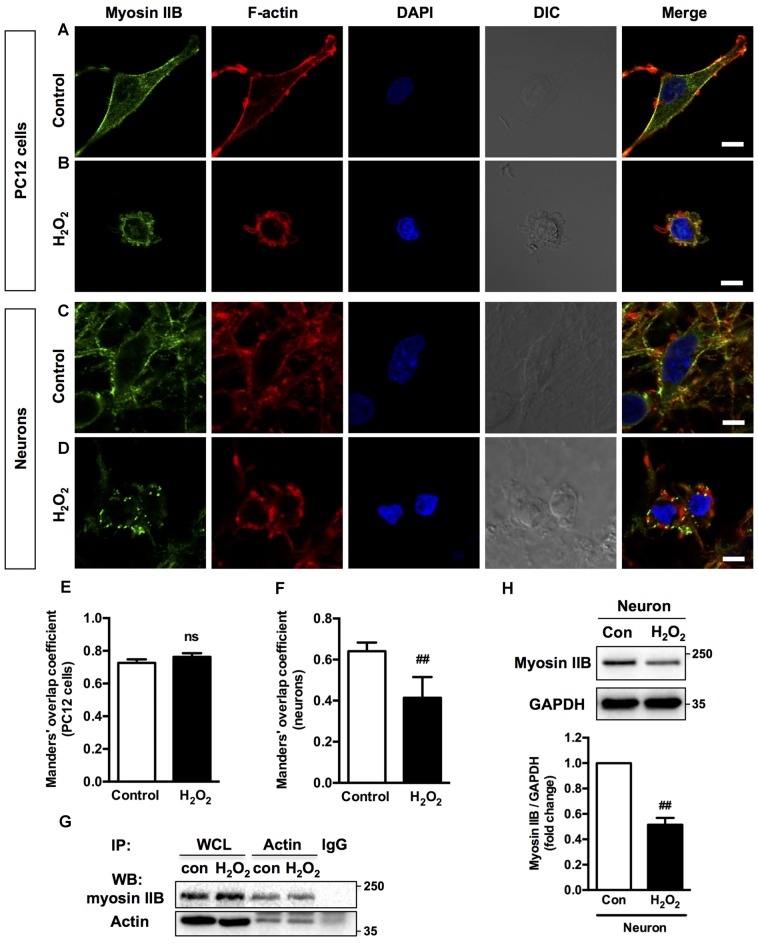
**Localization of myosin IIB and F-actin in PC12 cells or neurons upon H_2_O_2_ treatment.** PC12 cells untreated **(A)** or treated **(B)** with 100 μM H_2_O_2_ for 12 h were stained with myosin IIB (green), F-actin (red) and DAPI (blue). Neurons untreated **(C)** or treated **(D)** with 100 μM H_2_O_2_ for 12 h were stained with myosin IIB (green), F-actin (red) and DAPI (blue). Images were obtained by confocal microscopy. Bar, 5 μm. The co-localization of myosin IIB with F-actin in PC12 cells **(E)** or neurons **(F)** was evaluated on the basis of Manders’ overlap coefficients. Results were expressed as mean ± SD (^##^*P* < 0.01 vs. control). **(G)** Protein interaction between myosin IIB and F-actin was determined by co-immunoprecipitation. Following treatment, cell lysates were immunoprecipitated with anti-actin antibody and isotype-matched IgG control. Each precipitated sample was detected for the presence of myosin IIB and actin by immunoblot analysis using specific antibodies. WCL prior to the immunoprecipitation served as input controls. **(H)** Expression of Myosin IIB in neurons treated or untreated with H_2_O_2_ was evaluated by immunoblot analysis.

Previous studies have shown that morphological changes in apoptotic cells are dependent on actomyosin cytoskeleton remodeling (Wickman et al., 2013; Turney et al., 2016). To understand which myosin II isoform regulates neuronal apoptosis, we examined the relocalization of the two myosin II isoforms and actin filaments in response to H_2_O_2_. In normal PC12 cells and neurons, myosin IIA and IIB have distinct cellular localization pattern, while actin showed a similar pronounced peripheral localization. Myosin IIA was distributed throughout the cytoplasm and neuritis (Figures 2A,C), whereas myosin IIB tended to be broadly peripheral and associated more with actin comparing to myosin IIA (Figures 3A,C). H_2_O_2_ exposure induced dramatic changes of cell morphology, as well as the reorganization of myosin IIA, IIB and F-actin. Myosin IIA and F-actin accumulated to form a dense spherical network. Myosin IIA had a stronger association with actin filaments under oxidative stress than normal conditions (Figures 2B,D). Quantitive analysis by Manders’ overlap coefficients (Bolte and Cordelières, 2006) showed that myosin IIA and actin filaments exhibited statistically significant co-localization upon H_2_O_2_ treatment in both PC12 cells (Figure 2E) and neurons (Figure 2F). Co-immunoprecipitation analysis also confirmed the increased interaction of myosin IIA and F-actin induced by oxidative stress (Figures 2G,H).

H_2_O_2_ treatment didn’t significantly increase the interaction of myosin IIB with actin filaments in apoptotic PC12 cells (Figures 3B,E). Co-immunoprecipitation analysis also showed that the interaction between myosin IIB and actin didn’t change in PC12 cells induced by H_2_O_2_ (Figure 3G). While in neurons, myosin IIB staining became dimmer and punctate with decreased co-localization with actin under H_2_O_2_ exposure (Figures 3D,F). Western blot analysis showed that H_2_O_2_ treatment significantly decreased myosin IIB expression in neurons (Figure 3H), while had no effect on the protein level of myosin IIA (Supplementary Figure S1A). H_2_O_2_ also didn’t change the level of myosin IIA of IIB in PC12 cells (Supplementary Figure S1B). We then examined the expression levels of myosin II isoforms in PC12 cells and neurons. There was higher myosin IIA expression in PC12 cells and similar myosin IIB levels in both kinds of cells (Supplementary Figure S1C).

Although the co-localization of myosin IIB with actin filaments was more striking under normal conditions, myosin IIA tended to bind with more actin filaments under H_2_O_2_ treatment. The increased interaction between myosin IIA and actin suggested that actin polymerization might also play a role during H_2_O_2_ exposure. Therefore, we applied actin filament assembly inhibitor, cytochalasin D before H_2_O_2_ injury. Cytochalasin D inhibited PC12 cells membrane blebbing and caspase-3 activation induced by H_2_O_2_ (Supplementary Figure S2).

### Distinct Effects of Myosin IIA or IIB Knockdown on H_2_O_2_-induced PC12 Cells Apoptosis

**Figure 4:**
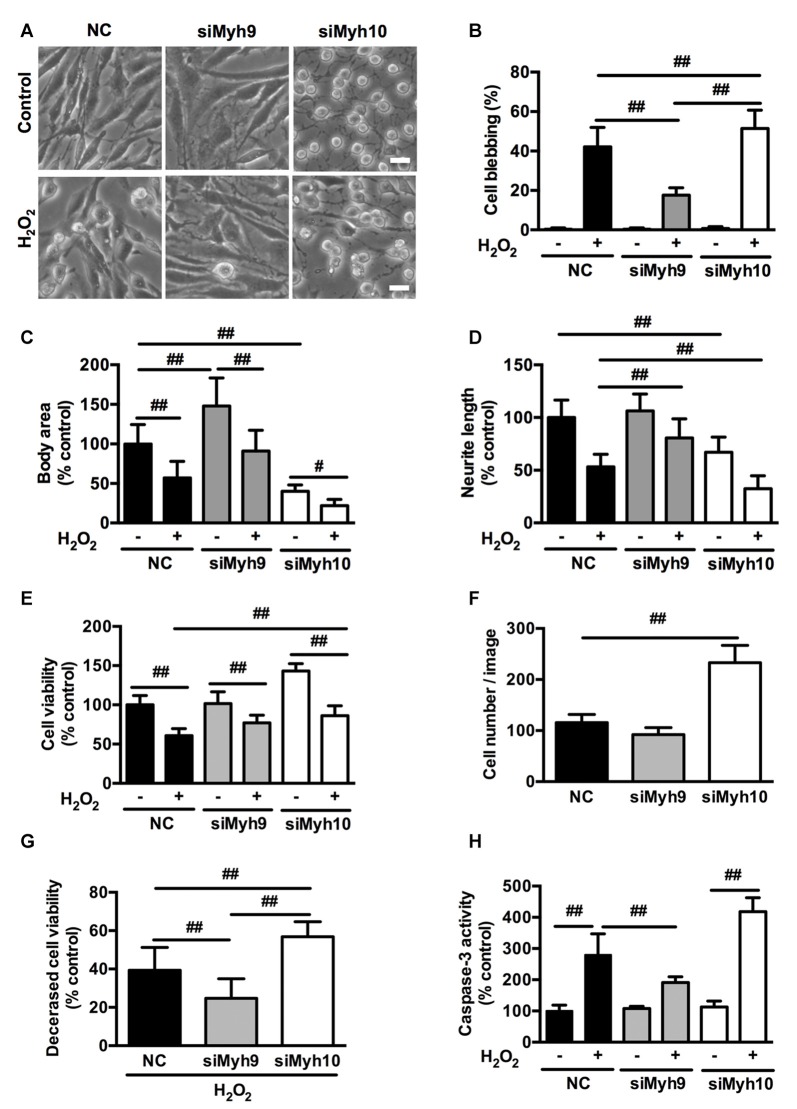
**Distinct effects of myosin IIA or IIB knockdown on H_2_O_2_-induced morphological changes and apoptosis in PC12 cells.** PC12 cells were transfected with siRNAs against myosin IIA (#1 siMyh9), myosin IIB (#1 siMyh10), or NC sequence as described in experimental procedures. **(A)** Representative phase-contrast micrographs of transfected PC12 cells treated or untreated with H_2_O_2_. Bar, 10 μm. **(B)** PC12 cells with membrane blebs were counted and the percentage of these cells with respect to the total cell population was calculated. Body area **(C)** and neurite length **(D)** was measured using the ImageJ software. The results were represented as percentage of control. **(E)** Viability of transfected cells was evaluated by 3-(4,5-dimethylthiazol-2-yl)-2,5-diphenyl tetrazolium bromide (MTT) assay. **(F)** The number of PC12 cells transfected with siRNAs targeting at myosin IIA or IIB was counted in the bright-field images. **(G)** Decreased cell viability was calculated by subtracting viability of H_2_O_2_-treated cells from control cells. **(H)** Caspase-3 activity was measured by caspase-3 activity assay kit. Values were represented as mean ± SD from three independent experiments (^#^*P* < 0.05, ^##^*P* < 0.01).

To investigate the specific roles of myosin II isoforms in neuronal apoptosis, we transfected PC12 cells with siRNAs targeting at myosin IIA or IIB. Transfection of PC12 cells with #1 *Myh9* siRNA (siMyh9) or 1# *Myh10* siRNA (siMyh10) led to attenuated expression of myosin IIA or IIB to 20%–30% of the control values, without affecting the levels of the other isoforms (Supplementary Figure S3). Knockdown of myosin IIA caused remarkable expansion in cell body area and increased neurite length. In contrast, depletion of myosin IIB led to a raised and rounded cell body with dramatic decreases in its size and neurite length, which suggests opposing roles of myosin IIA and IIB in regulating the morphology of PC12 cells (Figure 4D, Control). Knockdown of myosin IIA protected PC12 cells from H_2_O_2_-induced membrane blebbing, cell contraction and neurite retraction, compared with PC12 cells transfected with NC siRNA. In contrast, knockdown of myosin IIB further increased membrane blebbing, cellular contraction and neurite retraction under H_2_O_2_ exposure (Figures 4A–D, H_2_O_2_).

MTT assay showed that myosin IIA knockdown didn’t change, while myosin IIB knockdown increased cell viability in PC12 cells. Under H_2_O_2_ treatment, both myosin IIA and IIB knockdown increased cell viability compared with control (Figure 4E). This might result from the increased cell number following myosin IIB knockdown (Figure 4F). To exclude the effects of cell number, we then subtracted H_2_O_2_-induced cell viability from form untreated cells, which represents the decreased cell viability. There was a more severe reduction in cell viability of myosin IIB knockdown cells than that of control or myosin IIA knockdown cells (Figure 4G). Consistently, caspase-3 activity assay showed that myosin IIA depletion inhibited H_2_O_2_-induced caspase-3 activation, while myosin IIB knockdown further increased H_2_O_2_-induced caspase-3 activation in PC12 cells (Figure 4H). Our data indicated a critical role for myosin IIA in the progressive apoptosis of neuronal cells induced by H_2_O_2_.

### Myosin IIA Knockout by CRISPR/Cas9 Attenuates H_2_O_2_-induced PC12 Cells Apoptosis

**Figure 5:**
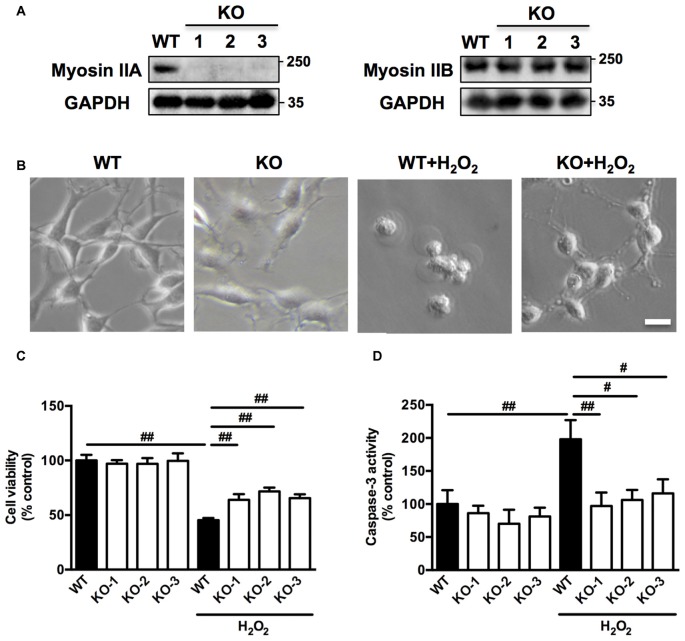
**Myosin IIA knockout by clustered regularly interspaced short palindromic repeats/CRISPR-associated protein-9 nuclease (CRISPR/Cas9) attenuates H_2_O_2_-induced morphological changes and apoptosis in PC12 cells. (A)** Stable *Myh9* knockout PC12 cells were established by CRISPR/Cas9. Cell lysates from single cell colonies #1, #2, #3 and wild-type (WT) cells were analyzed by western blotting using myosin IIA, myosin IIB and GAPDH antibodies. **(B)** The WT and *Myh9* knockout PC12 cells were untreated or treated with 100 μM H_2_O_2_ for 12 h. Cells were imaged using phase-contrast microscopy. Bar, 10 μm. **(C)** Viability of WT and *Myh9* knockout PC12 cells treated or untreated with H_2_O_2_ was evaluated by MTT assay. **(D)** Caspase-3 activity was measured by caspase-3 activity assay kit. Values were represented as mean ± SD from three independent experiments (^#^*P* < 0.05, ^##^*P* < 0.01).

To further confirm the role of myosin IIA in regulating H_2_O_2_-induced neuronal apoptosis, we knocked out *Myh9* gene in PC12 cells with the CRISPR/Cas9 system. Western blot analysis revealed that colony #1, #2 and #3 were completely depleted of myosin IIA protein compared with wild-type (WT) cells, without affecting the level of myosin IIB (Figure 5A). Consistently, *Myh9* knockout enlarged cell body area under normal conditions, and inhibited cell contraction and membrane blebbing upon H_2_O_2_ exposure (Figure 5B). *Myh9* knockout also increased cell viability and decreased caspase-3 activation after H_2_O_2_ treatment compared with WT cells (Figures 5C,D). Our observation demonstrates the critical and specific role of myosin IIA in regulating H_2_O_2_-induced PC12 cells apoptosis.

### H_2_O_2_ Induces Caspase-3/ROCK1/MLC Pathway Activation in Neuronal Cells

**Figure 6:**
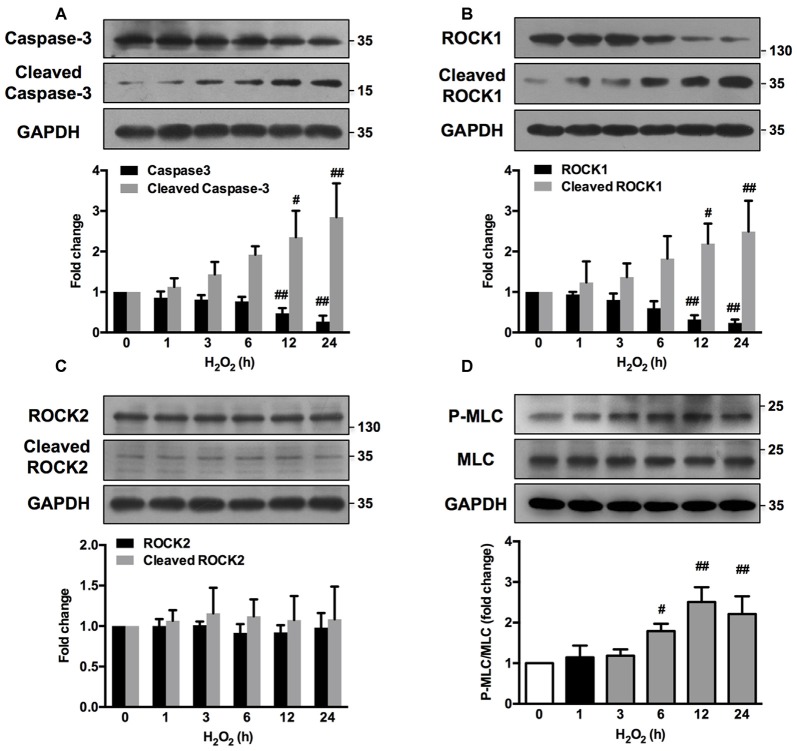
**H_2_O_2_ activates caspase-3/Rho-associated kinase (ROCK1)/myosin light chain (MLC) pathway in PC12 cells.** PC12 cells were incubated with 100 μM H_2_O_2_ for increasing periods of time. Total cell lysates were prepared, subjected to Western blot analysis with antibodies against caspase-3, cleaved caspase-3 **(A)**, ROCK1, cleaved ROCK1 **(B)**, ROCK2, cleaved ROCK2 **(C)**, MLC and P-MLC (Ser-19) **(D)**, and incubated with anti-GAPDH as loading control. Results were expressed as mean ± SD from three independent experiments (^#^*P* < 0.05, ^##^*P* < 0.01 vs. control).

ROCK1 contributes to actomyosin contractility and causes cellular blebs to protrude through MLC phosphorylation (P-MLC). Caspase-3 has also been reported to be important in such morphological changes (Sebbagh et al., 2001). Time-course study revealed that H_2_O_2_ increased the cleavage of caspase-3 as early as 3 h after H_2_O_2_ exposure (Figure 6A). A small cleavage fragment of Mr 30K ROCK1 was detectable using the C-19 antibody after 6 h of H_2_O_2_ treatment, while the abundance of the full-length Mr 160K protein decreased reciprocally (Figure 6B). In contrast, no such fragment or reduction of the Mr 160K band was observed using the specific anti-ROCK2 antibody (C-20; Figure 6C). MLC phosphorylation culminated after 12 h of H_2_O_2_ incubation and decreased thereafter (Figure 6D). Caspase-3, ROCK1, ROCK2 and MLC phosphorylation had similar changes in neurons upon H_2_O_2_ treatment for 12 h (Supplementary Figure S4). Together, there was a sequential activation of caspase3, ROCK1 and MLC during H_2_O_2_ exposure in neuronal cells.

### Caspase, ROCK or Myosin II Inhibition Attenuates H_2_O_2_-induced Neuronal Apoptosis and Membrane Blebbing

**Figure 7:**
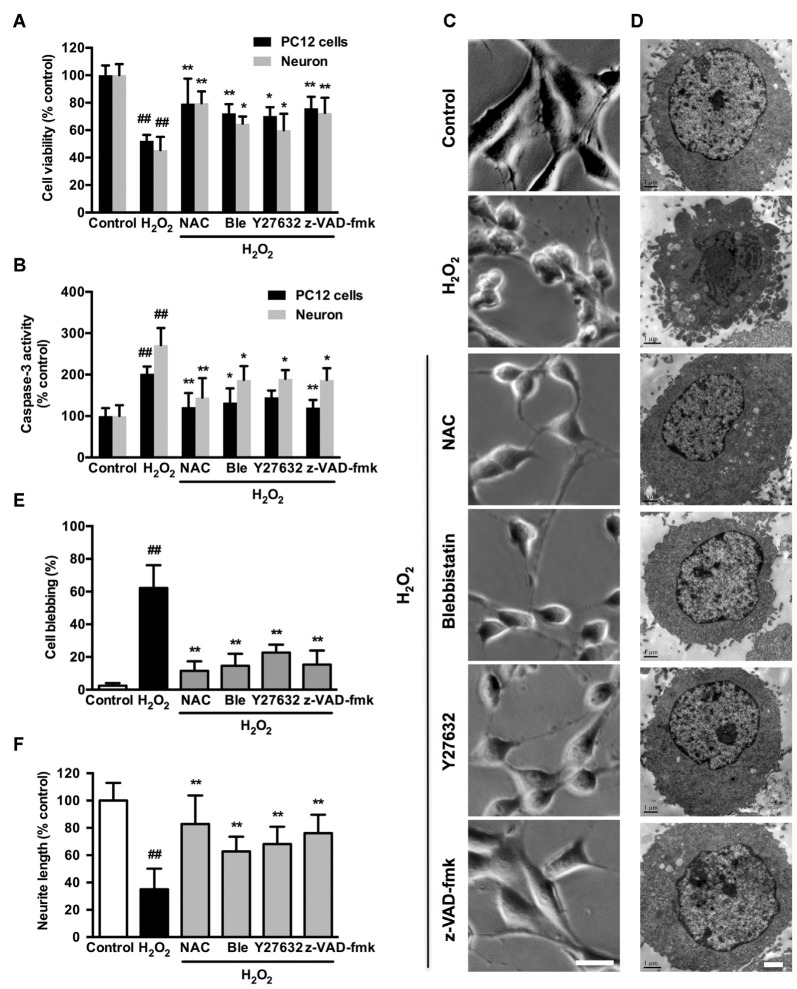
**Caspase, ROCK or myosin II inhibition attenuates H_2_O_2_-induced neuronal apoptosis, membrane blebbing and neurite retraction.** PC12 cells or neurons were untreated or pretreated with 1 μM blebbistatin, 10 μM Y27632 or 10 μM z-VAD-fmk for 1 h prior to 100 μM H_2_O_2_ treatment for 12 h. N-acetyl-L-cysteine (NAC; 500 μM) served as positive control. **(A)** Viability of PC12 cells and neurons was evaluated by MTT assay. **(B)** Caspase-3 activity of PC12 cells and neurons was determined by caspase-3 activity assay kit.** (C)** Representative phase-contrast micrographs of PC12 cells. Bar, 10 μm. **(D)** TEM of treated PC12 cells. Bar, 1 μm. **(E)** PC12 cells with membrane blebs were counted and the percentage of these cells with respect to the total cell population was calculated. **(F)** Neurite length was measured using the ImageJ software. The results were represented by the percentage of neurite length compared to control. Results were expressed as mean ± SD from three independent experiments (^##^*P* < 0.01 vs. control, *P < 0.05 vs. H_2_O_2_-treated cells, **P < 0.01 vs. H_2_O_2_-treated cells).

**Figure 8:**
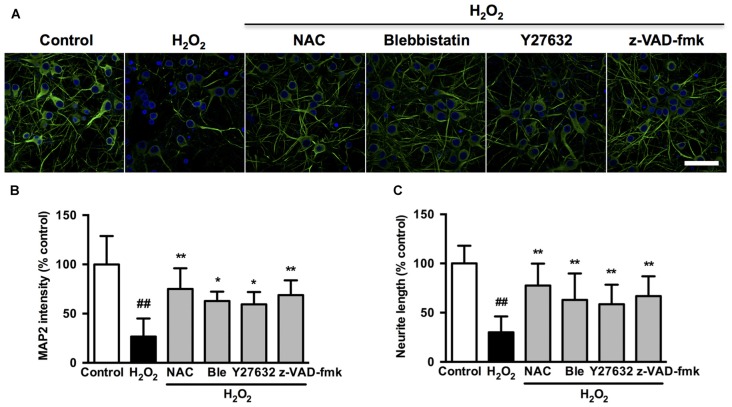
**Caspase, ROCK or myosin II inhibition decreases microtubule-associated protein 2 (MAP2) intensity and attenuates neurite retraction induced by H_2_O_2_ in neurons. (A)** Effects of blebbistatin, Y27632 and z-VAD-fmk on neuronal MAP2 fluorescence (green) after H_2_O_2_ treatment. **(B)** Quantification of MAP2 fluorescence intensity in treated neurons. Bar, 40 μm. **(C)** Neurite length of neurons was measured using the ImageJ software. The results were represented by percentage of neurite length compared to control. Results were expressed as mean ± SD from three independent experiments (^##^*P* < 0.01 vs. control, *P < 0.05 vs. H_2_O_2_-treated neurons, **P < 0.01 vs. H_2_O_2_-treated neurons).

We further investigated whether the observed association among caspase, ROCK or myosin II during apoptosis could be blocked by related chemical inhibitors. On the basis of cell viability, 1 μM blebbistatin was applied to reduce any possible toxic and nonspecific effects. Pretreatment with 1 μM blebbistatin (myosin II ATPase inhibitor), 10 μM Y27632 (ROCK inhibitor) or 10 μM z-VAD-fmk (caspase inhibitor) increased cell viability (Figure 7A), decreased caspase-3 activity (Figure 7B), inhibited membrane blebbing and increased neurite length (Figure 7C) after H_2_O_2_ exposure in both PC12 cells and neurons. Consistent with phase-contrast micrographs, TEM results indicated that pretreatment with blebbistatin, Y27632 and z-VAD-fmk blocked membrane blebbing and nucleus condensation induced by H_2_O_2_ in PC12 cells (Figure 7D). Statistical analysis showed that these inhibitors significantly decreased percentage of blebbing cells and increased neurite length after H_2_O_2_ treatment (Figures 7E,F). MAP2, a cytoskeletal phosphoprotein, is reported to degrade after ischemia and other injuries. Loss of MAP2 immunoreactivity is a marker of neuronal damage (Shelton et al., 2015). Blebbistatin, Y27632 and z-VAD-fmk rescued the decrease of MAP2 fluorescence and neurite length in neurons under H_2_O_2_ induction (Figure 8).

Caspase-3 inhibitor, z-DEVD-fmk inhibited H_2_O_2_-induced membrane blebbing and caspase-3 activity (Supplementary Figure S5), which indicated that caspase-3 activation played a major role in H_2_O_2_-induced apoptosis. MLC phosphorylation may occur via ROCK or MLCK. To address the role of MLCK in H_2_O_2_-induced membrane blebbing, cells were incubated with MLCK inhibitor ML-7 prior to H_2_O_2_ treatment. ML-7 failed to inhibit membrane blebbing and caspase3 activity (Supplementary Figure S5), which indicated that MLCK was probably not involved in H_2_O_2_-induced neuronal apoptosis.

### Caspase, ROCK or Myosin II Inhibition Attenuates H_2_O_2_-induced Caspase-3/ROCK1/MLC/Myosin IIA-actin Cascade Pathway Activation

**Figure 9:**
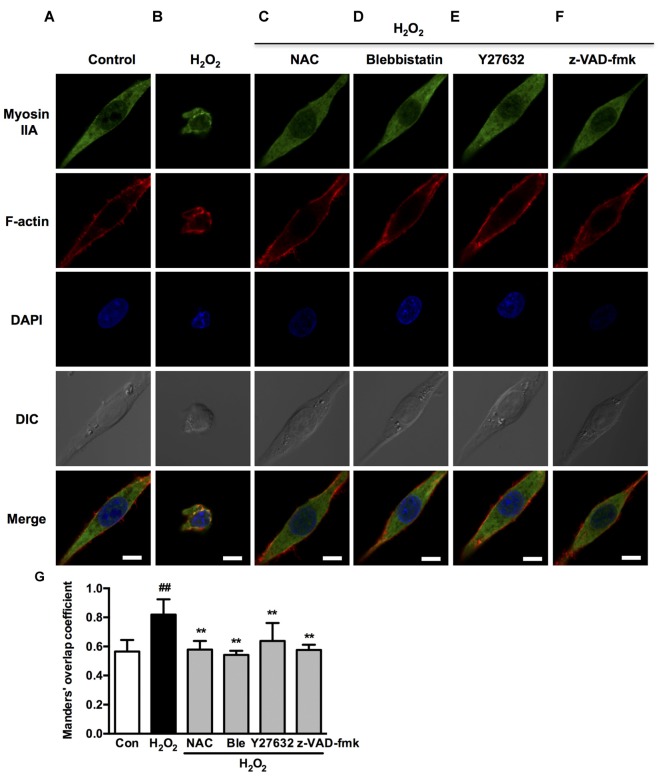
**Caspase, ROCK, myosin II inhibition attenuates H_2_O_2_-induced myosin IIA-actin interaction in PC12 cells. (A–F)** PC12 cells were untreated or pretreated with 1 μM blebbistatin, 10 μM Y27632 or 10 μM z-VAD-fmk for 1 h prior to 100 μM H_2_O_2_ treatment for 12 h. Positive control was treated with NAC (500 μM) for 1 h prior to H_2_O_2_ exposure. Myosin IIA (green), filamentous actin (red) and DAPI (blue) were detected by confocal microscope as indicated in Figure 2. Bar, 5 μm. **(G)** The quantitative co-localization of myosin IIA with F-actin was evaluated on basis of Manders’ overlap coefficients. Results were expressed as mean ± SD (^##^*P* < 0.01 vs. control, **P < 0.01 vs. H_2_O_2_-treated cells).

**Figure 10:**
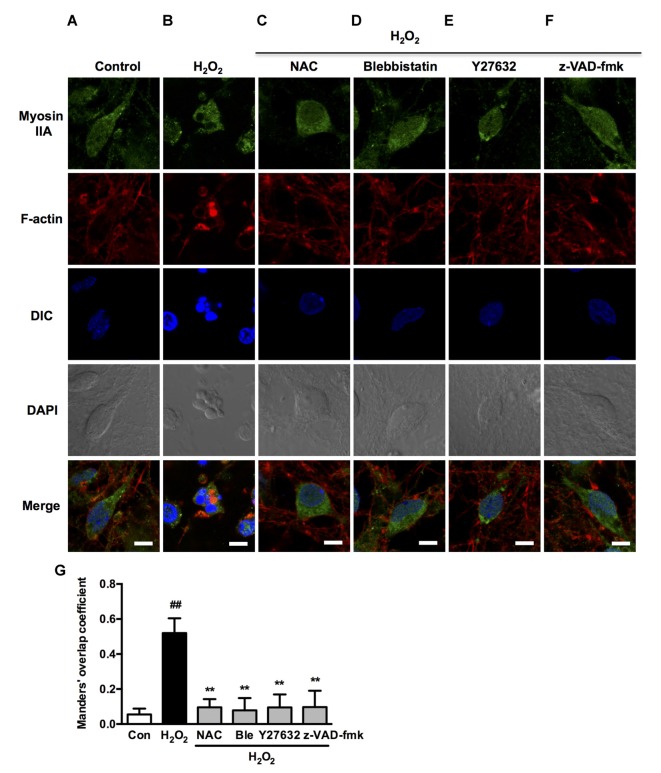
**Caspase, ROCK, myosin II inhibition attenuates H_2_O_2_-induced myosin IIA-actin interaction in neurons. (A–F)** Neurons were untreated or pretreated with 1 μM blebbistatin, 10 μM Y27632 or 10 μM z-VAD-fmk for 1 h prior to 100 μM H_2_O_2_ treatment for 12 h. NAC (500 μM) served as positive control. Myosin IIA (green), filamentous actin (red) and DAPI (blue) were detected by confocal microscope. Bar, 5 μm. **(G)** The quantitative co-localization of myosin IIA with F-actin was evaluated on the basis of Manders’ overlap coefficients. Results were expressed as mean ± SD (^##^*P* < 0.01 vs. control, **P < 0.01 vs. H_2_O_2_-treated cells).

We next investigated if blebbistatin, Y27632 or z-VAD-fmk attenuated myosin IIA-actin interaction induced by H_2_O_2_. In the presence of blebbistatin, Y27632 or z-VAD-fmk, the inhibition of membrane blebbing was associated with a reversion of the cell morphology and myosin IIA-actin reorganization toward control cells, exhibiting decreased myosin IIA-actin interaction (Figures 9A–F). The statistical analysis also revealed that blebbistatin, Y27632 or z-VAD-fmk significantly inhibited the co-localization between myosin IIA and actin filaments (Figure 9G). Similar results were also observed in neurons (Figure 10). These findings demonstrated that caspase-3/ROCK1/MLC activation plays a key role in triggering the downstream myosin IIA-actin hyperactivation upon H_2_O_2_ exposure.

**Figure 11:**
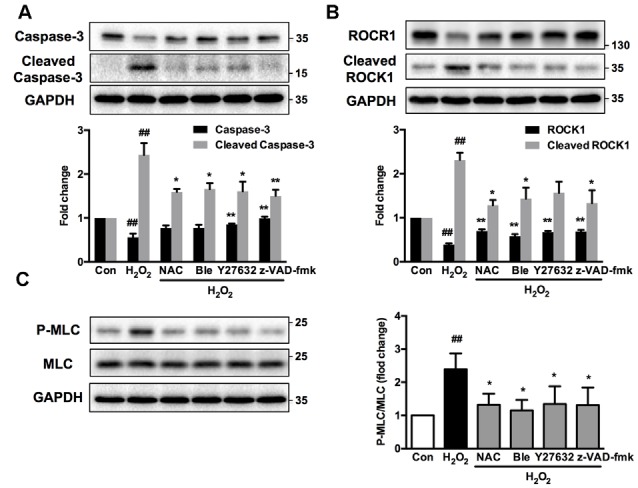
**Caspase, ROCK, myosin II inhibition attenuates H_2_O_2_-induced signaling pathway activation in PC12 cells.** PC12 cells were untreated or pretreated with 1 μM blebbistatin, 10 μM Y27632 or 10 μM z-VAD-fmk for 1 h prior to 100 μM H_2_O_2_ treatment for 12 h. NAC served as positive control. Total cell lysates were prepared, subjected to Western blot analysis with antibodies to caspase-3, cleaved caspase-3 **(A)**, ROCK1, cleaved ROCK1 **(B)**, MLC and P-MLC (Ser-19) **(C)**, and incubated with anti-GAPDH antibody as loading control. Results were expressed as mean ± SD from three independent experiments (^##^*P* < 0.01 vs. control, *P < 0.05 vs. H_2_O_2_-treated cells, **P < 0.01 vs. H_2_O_2_-treated cells).

**Figure 12:**
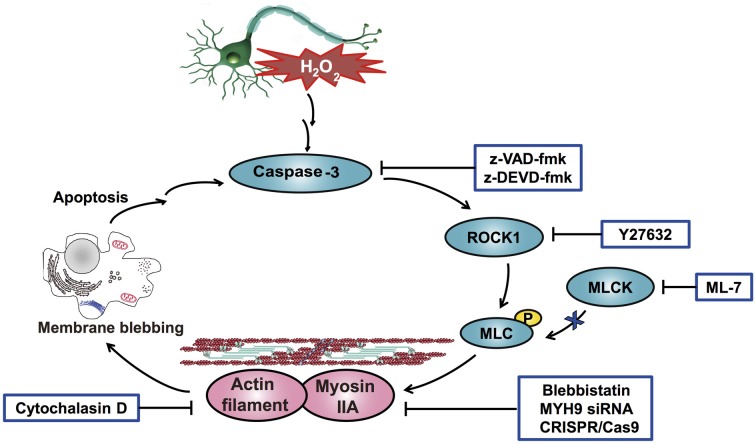
**Schematic overview of the proposed mechanism for H_2_O_2_-induced neuronal apoptosis.** H_2_O_2_ induces caspase-3 activation through upstream signaling pathways and then stimulates the cleavage of ROCK1, which promotes the phosphorylation of MLC (Ser-19). Phosphorylated MLC activates myosin IIA and facilitates its association with actin filaments. Consequently, the increased interaction of myosin IIA and actin generates the actomyosin contractility, which initiates membrane blebbing and neuronal apoptosis. In addition, the increased interaction of myosin IIA and actin amplifies and sensitizes H_2_O_2_-induced apoptosis. Collectively, a positive feedback loop of caspase-3/ROCK1/MLC/myosin IIA-actin contractility mediates H_2_O_2_-induced neuronal apoptosis.

Interestingly, Western blot analysis showed that myosin IIA-actin interaction inhibition by blebbistatin attenuated H_2_O_2_-induced activation of caspase-3, ROCK1 and MLC, indicating that myosin IIA might reversely influence caspase-3, ROCK1 and MLC activity. Y27632 and z-VAD-fmk also attenuated H_2_O_2_-induced cleaved caspase-3, cleaved ROCK1 and MLC phosphorylation (Figure 11). These results demonstrated that H_2_O_2_-induced caspase-3/ROCK1/MLC activation and myosin IIA-actin interaction cascade might form a positive feedback pathway in H_2_O_2_-induced neuronal apoptosis (Figure 12). The original uncropped images of western blots are shown in Supplementary Figures S6–S9.

## Discussion

### Interaction of Myosin IIA or IIB with Actin Filaments in Neuronal Cells

The interaction of myosin II with actin filaments in generating forces during the execution phase of apoptosis has been well documented (Ndozangue-Touriguine et al., 2008). However, little is known about the specific functions of myosin II isoforms in neuronal apoptosis. Normally, myosin IIA was dispersed throughout the cytoplasm, in contrast to the pronounced peripheral localization of myosin IIB and F-actin, which indicated that myosin IIA was better suited for large scale contractile processes than myosin IIB (Wylie and Chantler, 2003). Accordingly, the interaction of myosin IIA and actin increased upon H_2_O_2_ treatment, whereas myosin IIB and actin interaction showed no obvious change in PC12 cells and decreased in neurons as myosin IIB protein degraded. Why was myosin IIA retained but IIB lost after H_2_O_2_ exposure in neurons? On one hand, our results have demonstrated the requirement of actin filaments for cell contraction and membrane bleb formation during neuronal apoptosis. On the other hand, the interaction of myosin IIB with actin filaments was more striking compared with that of myosin IIA under normal conditions. Therefore, decreased myosin IIB protein level could facilitate the binding between myosin IIA and actin filaments due to lack of competing with myosin IIB in neurons. Although myosin IIB levels were virtually not affected in PC12 cells, myosin IIB is reported to be less active than myosin IIA (Kelley et al., 1996), and bind with actin more slowly than myosin IIA (Kolega, 2003, 2006). Therefore, the differential localization and expression of myosin IIA and IIB, together with the spatial organization of actin filaments are critical to their actions (Beach and Hammer, 2015).

### Distinct Roles of Myosin IIA and IIB in Neuronal Cells

Cellular activity, like growth and motility, is the resultant of a balance of forces, the mechanism involving two opposing processes: outgrowth and retraction. Each of these processes is regulated by the coordinated actions of one or more molecular motors (Chantler and Wylie, 2003; Hyland et al., 2014). It has reported that myosin IIA drives neurite retraction and maintains tensile adhesion (Wylie and Chantler, 2001), while myosin IIB drives outgrowth (Wylie et al., 1998). Knockdown of myosin IIA or IIB resulted in opposite morphological changes. Thus, the functions of myosin IIA and IIB counteracted each other. Our data indicated that loss of myosin IIA would result in a shift in the balance of myosin IIB-based outgrowth, while loss of myosin IIB resulted in the myosin IIA-mediated retraction. As myosin IIA derived large scale cellular contractile processes under oxidative stress-induced apoptosis, myosin IIB knockdown further increased the interaction of myosin IIA with actin and the related contractile forces generation, which caused cells more sensitive to oxidative stress and easier to undergo apoptosis.

It has been reported that myosin IIA depletion remarkably increases cell survival and cloning efficiency of human/mouse embryonic stem cells (h/mESCs; Walker et al., 2010). Myosin IIB, together with actin, constitute the actomyosin cytoskeleton that mediates contractility during TNFalpha-induced shrinkage, detachment and apoptosis in NIH 3T3 fibroblasts (Solinet and Vitale, 2008). Our results demonstrated that myosin IIA knockdown protected cells from cellular contraction, caspase-3 cleavage and apoptosis. We also showed for the first time that PC12 cells with myosin IIB knockdown had a higher proliferative activity than myosin IIA knockdown or control cells. It has been reported that cytokinesis is facilitated by the constriction of a cortical ring made up of myosin II and actin filaments in animal and fungal cells (Cheffings et al., 2016; Zambon et al., 2017). Actomyosin-drived cellular contractility is recognized as an important promoter for increased tumor number, growth and progression (Samuel et al., 2011). And myosin IIA stack occurs in the cleavage furrow of dividing cells and form the contractile ring to divide cells (Fenix et al., 2016). Therefore, myosin IIB depletion might facilitate the myosin IIA-mediated cellular contractility that is essential for cytokinesis, causing increased PC12 cell number. These results indicated that myosin II regulates cell survival in an isoform and cell type specific manner.

Such an antagonistic relationship between these two motors on the morphology of PC12 cells and responses to oxidative stress may be an important mechanism by which the vectorial balance of forces is regulated during cellular growth and apoptosis. Myosin IIA knockout by CRISPR/Cas9 provides evidence of a central role for myosin IIA in cellular contractility-induced apoptosis under oxidative stress. This work also highlights the use of the CRISPR/Cas9 to study neuroscience and cytoskeletal functions.

### Oxidative Stress-induced Neuronal Apoptosis is Dependent on Actomyosin Contractility

Activation of the myosin II motor through ATP hydrolysis and binding to actin filaments are required to initiate actomyosin contractility, which further facilitates cellular apoptosis (Sari-Hassoun et al., 2016). Blebbistatin, a small molecular myosin inhibitor, exhibits high affinity and selectivity toward myosin II (Limouze et al., 2004; Allingham et al., 2005). Kinetic analysis shows that in the concentration range 0.5–5 μM, the inhibitor preferentially binds to the myosin-ADP-Pi complex and slows down phosphate release process. Thus, the inhibitor blocks the myosin II heads to interact with actin (Kovács et al., 2004). Several studies indicate the roles of blebbistatin in protection of damaged mammalian cells. Blebbistatin inhibits TNFα/CHX induced apoptotic nuclear breakdown and membrane blebbing in fibroblasts (Croft et al., 2005). Inhibiting myosin II activity by blebbistatin markedly promotes growth cone invasion and accelerates axon growth rate on inhibitory substrates (Burnette et al., 2007; Hur et al., 2011). Although blebbistatin potently inhibits actin-myosin interaction, very little is known regarding its action in the organized contractile system of oxidative stress-induced neuronal apoptosis. Myosin II uses ATP to move along actin filaments, which forms actomyosin system in various cellular functions (Hartman and Spudich, 2012). Cytochalasin D, a blocker of actin polymerization, disruptes actin filaments dynamics (Nair et al., 2008). Cytochalasin D treatment resists serum starvation-induced caspase-3 activation in NIH 3T3 cells by extracellularly activating gelatinase A, which interacts with integrin aVh3, eliciting survival signals mediated through ERK 1/2, p38 and SAPK/JNK pathways (Ailenberg and Silverman, 2003). Consistently, both blebbistatin and cytochalasin D prevented H_2_O_2_-induced activation of caspase-3, membrane blebbing and neurite retraction in neuronal cells. Our data demonstrate that the function of activated myosin II during neuronal apoptosis is associated with actin filament formation and actomyosin contractility.

### Myosin IIA-actin Interaction is involved in a Positive Feedback Loop with Caspase-3/ROCK1/MLC Phosphorylation

Exogenous H_2_O_2_ influences the function of various proteins, such as kinases, phospholipases, transcription factors, ion channels, G proteins, et al. However, unlike other second messengers, the structure of H_2_O_2_ is too small and simple to interact with a specific protein target (Rhee et al., 2003). It has been reported that H_2_O_2_ induces neuronal apoptosis through various signaling pathway, such as nuclear factor-κB, JNK/ERK (Liu et al., 2017), Cdc42/MLK3/MKK7/JNK3 pathway (Wang et al., 2017), P53, mitochondria-related Bax and Bcl-2 (Sohn et al., 2016), Akt/H2A phosphorylation (Park et al., 2016), et al. In out research, time-course study revealed that H_2_O_2_ activated caspase-3, ROCK1 and MLC in chronological sequence. Thus, H_2_O_2_ activated caspase-3 through upstream pathways and then activated caspase-3 mediated ROCK1 and MLC activation. However, the upstream signaling of caspase-3 still needs to be further investigated.

It has been previously demonstrated that caspase/ROCK-mediated MLC phosphorylation and the mitochondrial signaling axis regulate cell apoptosis (Liu et al., 2013; Shen et al., 2015b). Our findings extended previous observations by showing that activated caspase-3, ROCK1 and phosphorylated MLC were specifically associated with the myosin IIA-actin cytoskeletal system. Disruption of myosin IIA-actin contraction is the mechanism by which caspase or ROCK inhibitors decreased apoptosis and membrane blebbing of neuronal cells. Interestingly, inhibition of myosin IIA-actin contraction by blebbistatin, myosin IIA knockdown, knockout or cytochalasin D in turn inhibited caspase-3 activation. Blebbistatin also inhibited ROCK1 activation and MLC phosphorylation, which was similar with the effects of Y27632 and z-VAD-fmk. These results indicated that myosin IIA and its interaction with actin filaments could regulate caspase-3/ROCK1/MLC phosphorylation in a positive feedback manner, which has numerous implications. Once activated, this positive feedback loop may be an important mechanism to amplify and sustain actomyosin contractility, signaling cascade activation and ultimate apoptosis. This architecture also explains the observation that disruption of caspase, ROCK, myosin IIA or actin functionally disrupted the entire mechanical system and signaling pathway, resulting in similar phenotypes such as decreased membrane blebbing, apoptosis and neurite retraction.

It has been suggested an existence of a feedback loop between cytoskeletal tension, adhesion maturation and ROCK signaling that contributes to numerous mechanochemical processes (Bhadriraju et al., 2007). Under the treatment of rotenone, PUMA, Bcl-XL and p53 form a positive feedback amplification loop to increase the apoptosis sensitivity (Shi et al., 2014). Additionally, downstream caspase-3 reinforces Bak activation and the release of AIF and endoG through positive loops, which sensitize cancer cells to the treatment with genistein (Guo et al., 2015). The numerous feedback loops between cytoskeletal, apoptotic and mechanical functions of cells and the related signaling pathway highlight the complexity of pathological and physiological mechanisms in cells. Other studies have also reported that myosin IIA knockdown or blebbistatin pretreatment remarkably increase cell survival and cloning efficiency of embryonic stem cells (Walker et al., 2010). Moreover, inhibition of actin polymerization by cytochalasin D inhibits thrombin-induced activation of caspase-3 and caspase-9 in human platelets (Ben Amor et al., 2006). However, how the cellular contractile forces regulate caspase-3 activity has not been fully elucidated. Investigating these mechanisms will be important to us to understand how cells coordinate the mechanical and biochemical events.

### Future Direction of the Research

As our data support a regulatory loop between myosin IIA and caspase-3/ROCK1/MLC pathway, this raises the question about how the cell specifically regulates myosin IIB activity. It has been shown that myosin IIB is activated upstream by PKC, PAK1 or Rac, which have been shown to regulate the phosphorylation and cellular localization of myosin IIB and induce neurite outgrowth (Even-Faitelson et al., 2005; Solinet and Vitale, 2008). Regulation of myosin IIA and IIB by distinct signaling pathways might be an explanation for myosin II isoforms performing different functions at different places in cells. Clearly, additional work is required to fully understand the specific roles of myosin IIA and IIB, and how the two isoforms are distinctly regulated. In addition, myosin IIA and IIB share 77% identity at the amino acid level, they are believed to have considerable functional overlap in certain circumstances (Bao et al., 2007). Despite our observations that myosin IIA and IIB perform unique cellular functions, it still needs further research on the complementary actions and how the two isoforms influence each other in regulating neuronal function and apoptosis.

In conclusion, we identified the distinct functions of the two highly conserved myosin II motors in oxidative stress-induced neuronal apoptosis. Myosin IIA, rather than myosin IIB, constitutes a continuous mechanical link with actin filaments, and is required to develop contractile forces and membrane blebbing during H_2_O_2_-induced neuronal apoptosis. Myosin IIA is also involved in a positive feedback loop that links caspase-3/ROCK1/MLC signaling axis. Understanding the cytoskeletal basis of neuronal cells would be helpful to clarify the cellular mechanisms underlying the biochemical, mechanistic and morphological changes induced by oxidative stress, and to develop therapeutics for neuronal diseases.

## Author Contributions

JK conceived and supervised the study. YW and JK designed experiments. YW and YX performed the experiments. YZ, GC, MY, NJ and BY provided facilities. YW, YX, YZ, BY, ZC and JK analyzed and interpreted the data. YW, QL and ZG wrote and revised the manuscript. ZC and JK reviewed the manuscript.

## Funding

This work was supported by the National Natural Science Foundation of China (No. 81274004) and the Fundamental Research Funds for the Central Universities (No. 2410724), a project funded by the Priority Academic Program Development of Jiangsu Higher Education Institution.

## Conflict of Interest Statement

The authors declare that the research was conducted in the absence of any commercial or financial relationships that could be construed as a potential conflict of interest.

## Abbreviations

CNS: central nervous system
CRISPR/Cas9: clustered regularly interspaced short palindromic repeats/CRISPR-associated protein-9 nuclease
DMSO: dimethyl sulfoxide
H_2_O_2_: hydrogen peroxide
MAP2: microtubule-associated protein 2
MHC: myosin heavy chain
MLC: myosin light chain
Myosin II: non-muscle myosin II
MLCK: myosin light chain kinase
MLCP: myosin light chain phosphatase
MTT: 3-(4,5-dimethylthiazol-2-yl)-2,5-diphenyl tetrazolium bromide
NAC: N-acetyl-L-cysteine
ROCK: Rho-associated kinase
TEM: transmission electron microscopy.
