# Supplementary material for: Myosin IIA-related Actomyosin Contractility Mediates Oxidative Stress-induced Neuronal Apoptosis
Source: Front Mol Neurosci. 2017 Mar 14;10:75. doi: 10.3389/fnmol.2017.00075 (PMC5348499; doi:10.3389/fnmol.2017.00075)
Supplement: Supplementary file 1 [file DataSheet_1.docx]

Supplementary Material

Myosin IIA-related Actomyosin Contractility Mediates Oxidative Stress-induced Neuronal Apoptosis

**Yan Wang^1^, Yingqiong Xu^1^, Qian Liu^2^, Yuanyuan Zhang^1^, Zhen Gao^3^, Mingzhu Yin^4^, Nan Jiang^1^, Guosheng Cao^1^, Boyang Yu^1^, Zhengyu Cao^1*^, Junping Kou^1*^**

*** Correspondence:**

Dr. Junping Kou

[junpingkou@cpu.edu.cn](mailto:junpingkou@cpu.edu.cn)

Dr. Zhengyu Cao

[zycao1999@hotmail.com](mailto:zycao1999@hotmail.com)

**
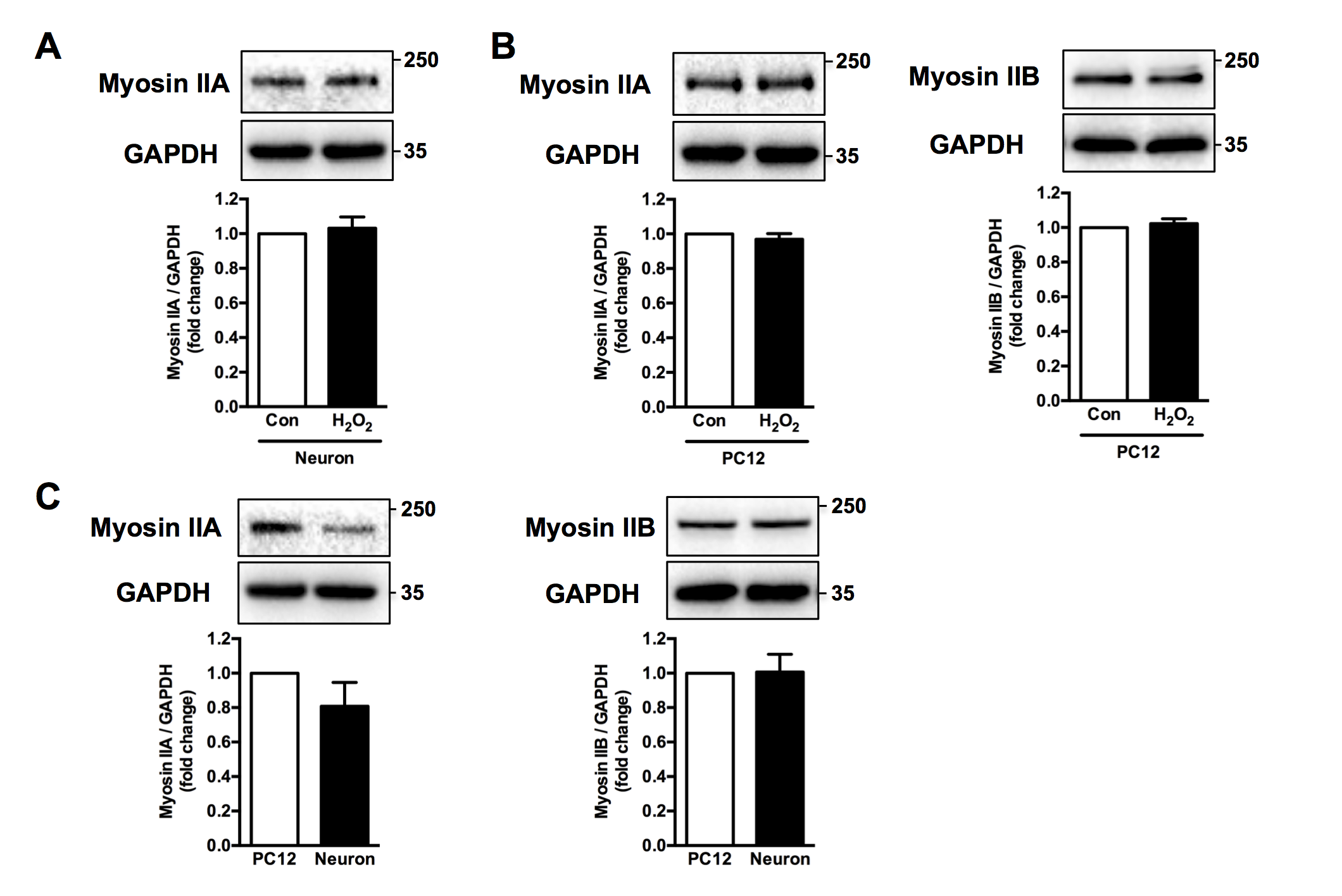
**

**Supplementary Figure S1. Expression of myosin IIA and IIB in PC12 cells and neurons treated or untreated with H_2_O_2_. (A)** Expression of Myosin IIA in neurons treated or untreated with H_2_O_2_. **(B)** Expression of Myosin IIA or IIB in PC12 cells treated or untreated with H_2_O_2_. **(C)** Expression of myosin IIA and IIB in PC12 cells and neurons.


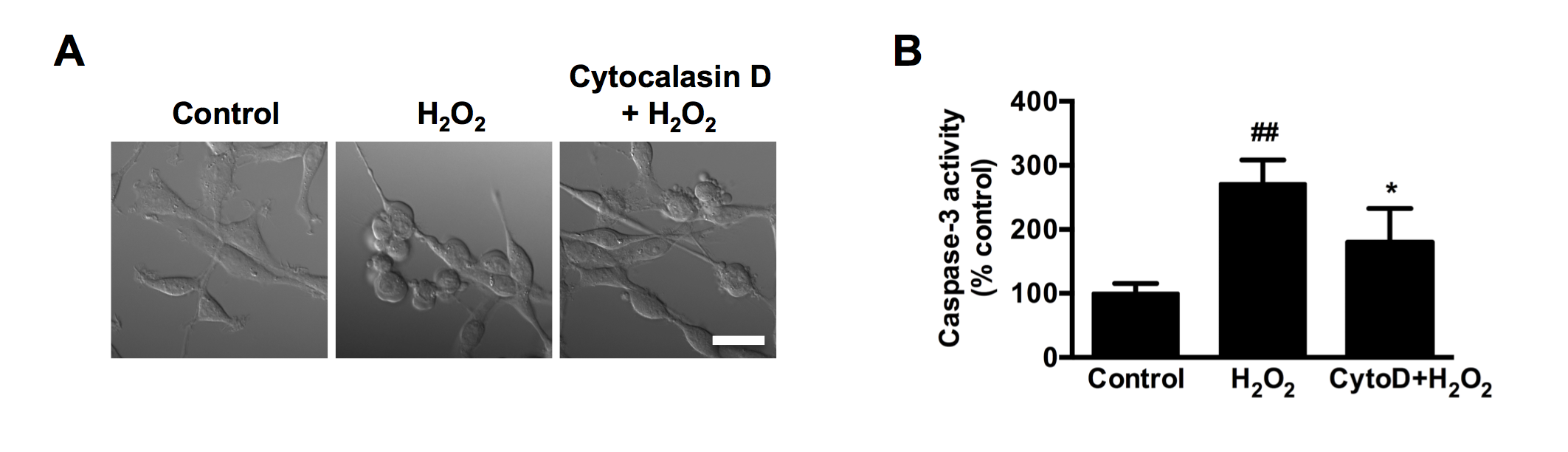


**Supplementary Figure S2. Cytochalasin D inhibits H_2_O_2_-induced membrane blebbing and caspase-3 activation in PC12 cells.** PC12 cells were pre-incubated with 1 μM cytochalasin D for 1 h, and then treated with 100 μM H_2_O_2_ for another 12 h. **(A)** Images of treated PC12 cells were taken through DIC using confocal laser scanning microscope. Bar, 20 μm. **(B)** Caspase-3 activity of treated PC12 cells. Values were represented as mean ± SD from three independent experiments (^##^*P* < 0.01 versus control, ^*^*P* < 0.05 versus H_2_O_2_-treated cells).


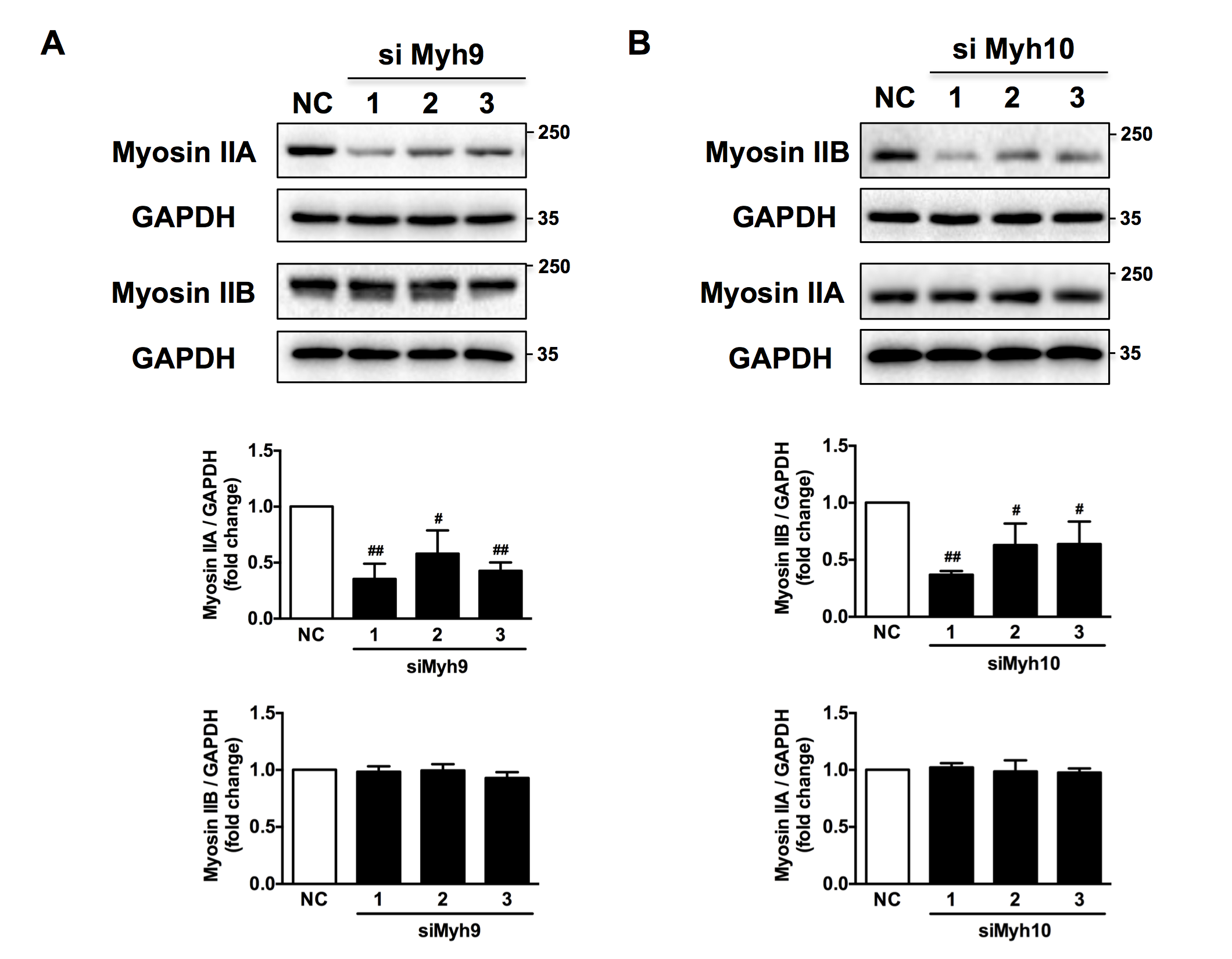


**Supplementary Figure S3. Myosin IIA or IIB knockdown in PC12 cells.** PC12 cells were transfected with negative control sequence (NC), three siRNAs against myosin IIA (siMyh9) or myosin IIB (siMyh10) to identify the most appropriated siRNAs, as described in experimental procedures. **(A-B)** Western blot analysis was performed on cell lysates collected 48 h after transfection to determine myosin IIA or IIB expression. Values were represented as mean ± SD from three independent experiments (^#^*P* < 0.05 versus control, ^##^*P* < 0.01 versus control).

**
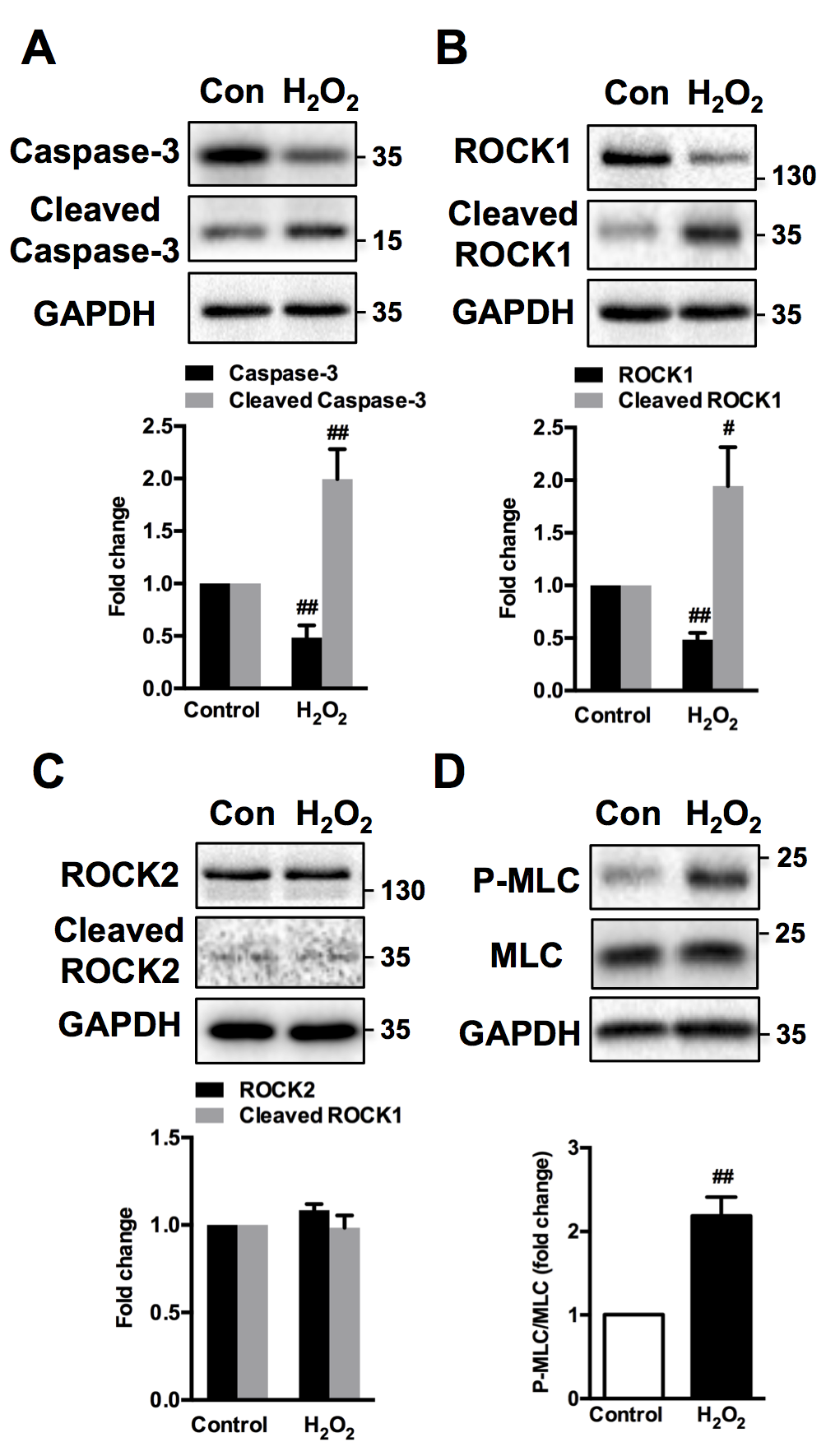
**

**Supplementary Figure S4. H_2_O_2_ induces caspase-3/ROCK1/MLC pathway activation in neurons.** Neurons were incubated with 100 μM H_2_O_2_ for 12 h. Total cell lysates were subjected to Western blot analysis with antibodies against caspase-3, cleaved caspase-3 **(A)**, ROCK1, cleaved ROCK1 **(B)**, ROCK2, cleaved ROCK2 **(C)**, MLC and P-MLC (Ser-19) **(D)**, and incubated with anti-GAPDH as loading control. Independent experiments were performed three times (^#^*P* < 0.05 versus control, ^##^*P* < 0.01 versus control).

**
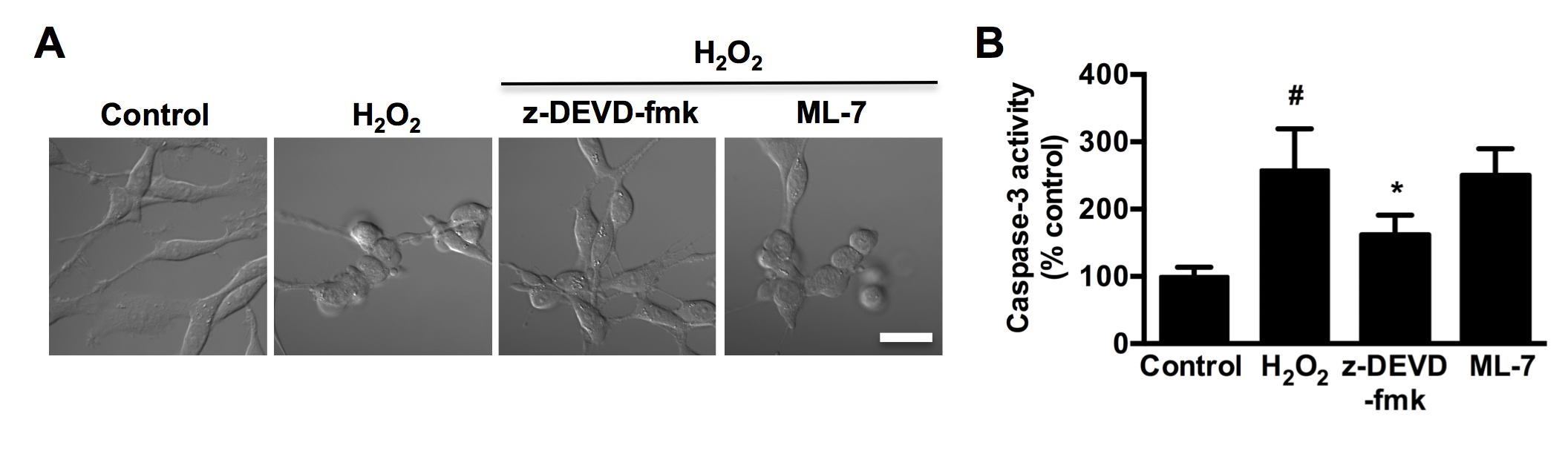
**

**Supplementary Figure S5. Effects of z-DEVD-fmk and ML-7 on H_2_O_2_-induced membrane blebbing and caspase-3 activation in PC12 cells.** PC12 cells were pre-incubated with 10 μM z-DEVD-fmk or 5 μM ML-7 for 1 h and then co-incubated with 100 μM H_2_O_2_ for another 12 h. **(A)** Images of treated PC12 cells were taken through DIC using confocal laser scanning microscope. Bar, 20 μm. **(B)** Caspase-3 activity of treated cells. Values were represented as mean ± SD from three independent experiments (^#^*P*  < 0.05 versus control, ^*^*P*  < 0.05 versus H_2_O_2_-treated cells).

**
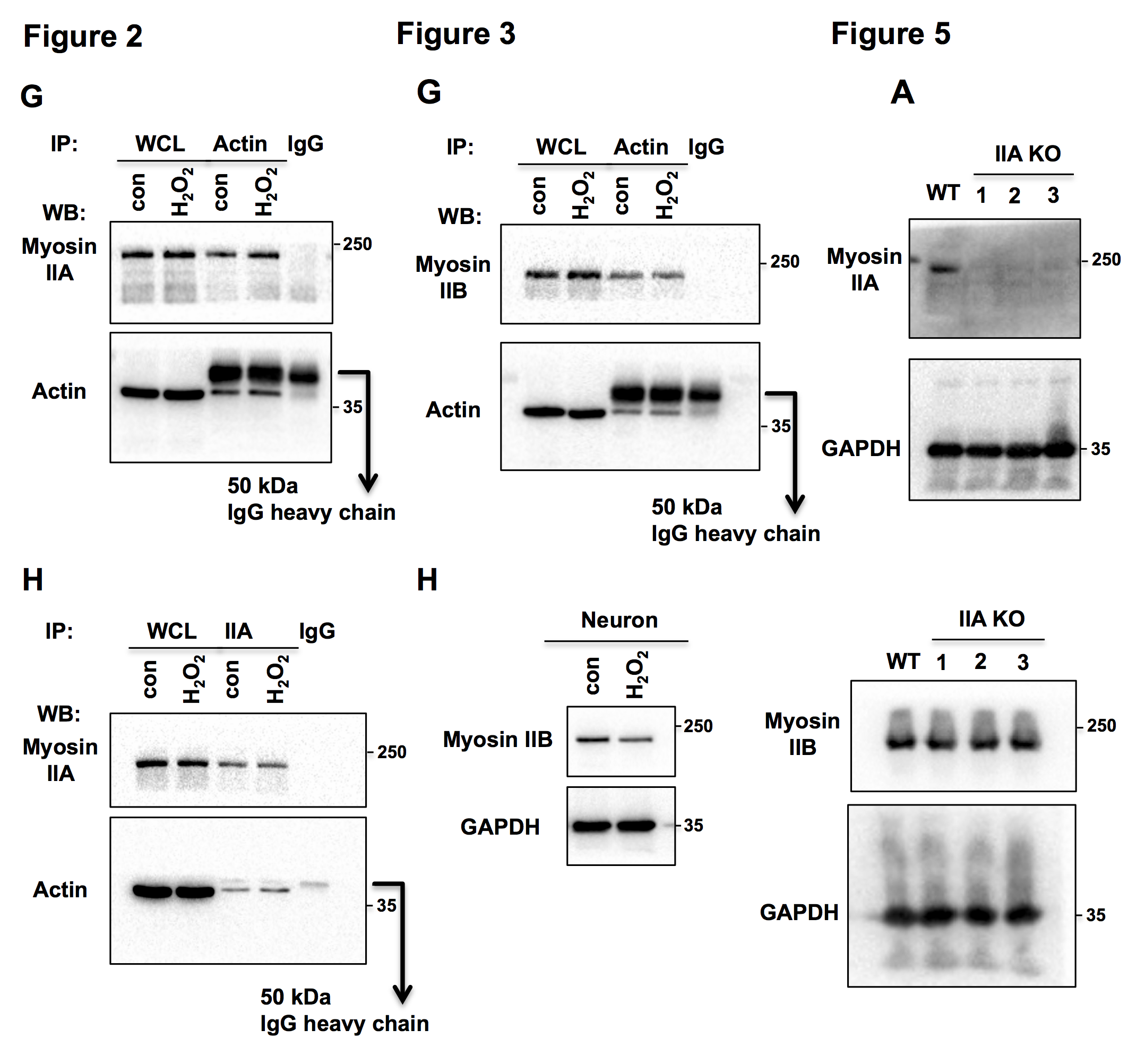
**

**Supplementary Figure S6. Uncropped Western Blot images of figure 2, 3 and 5.**

**
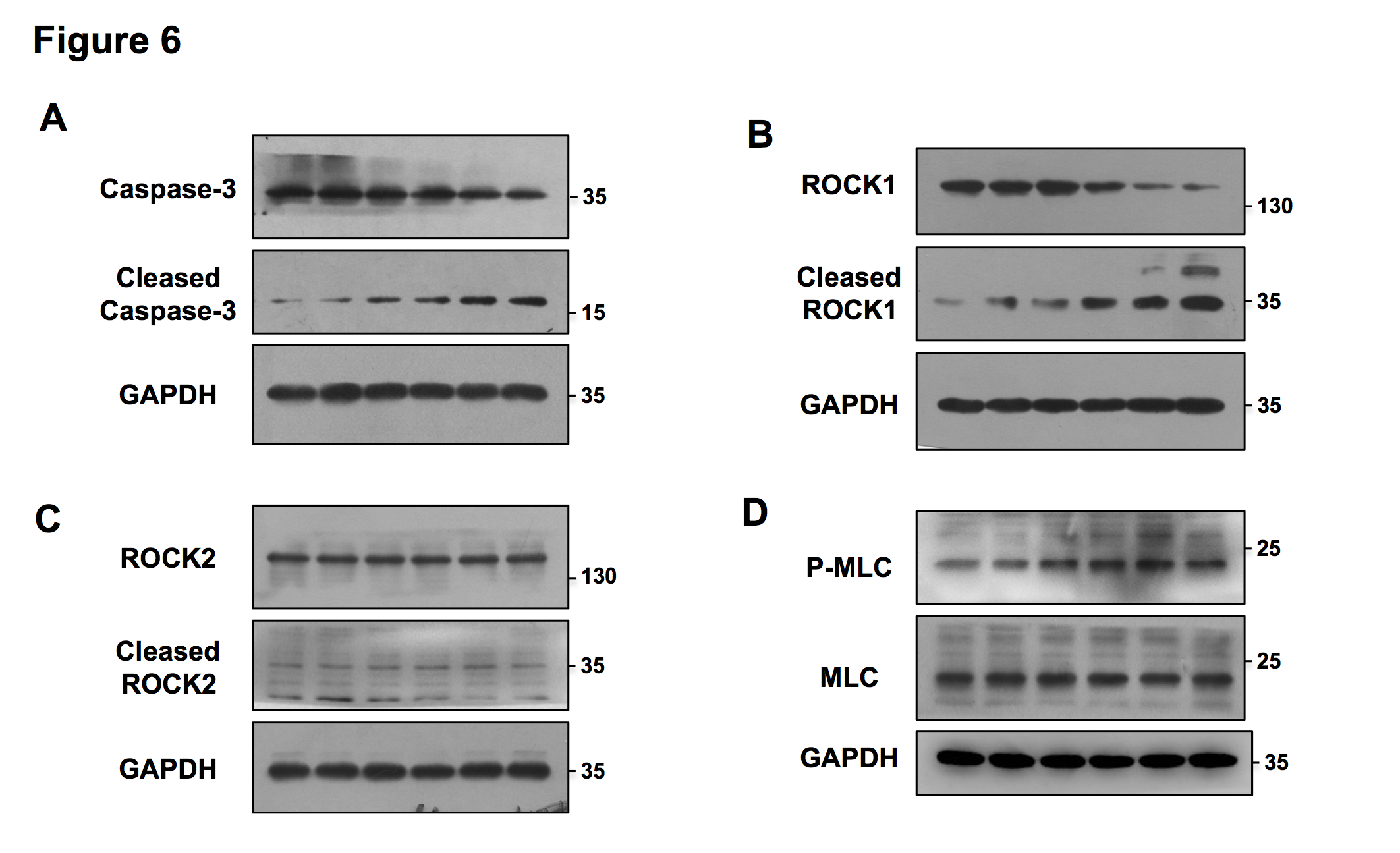
**

**Supplementary Figure S7. Uncropped Western Blot images of figure 6.**

**
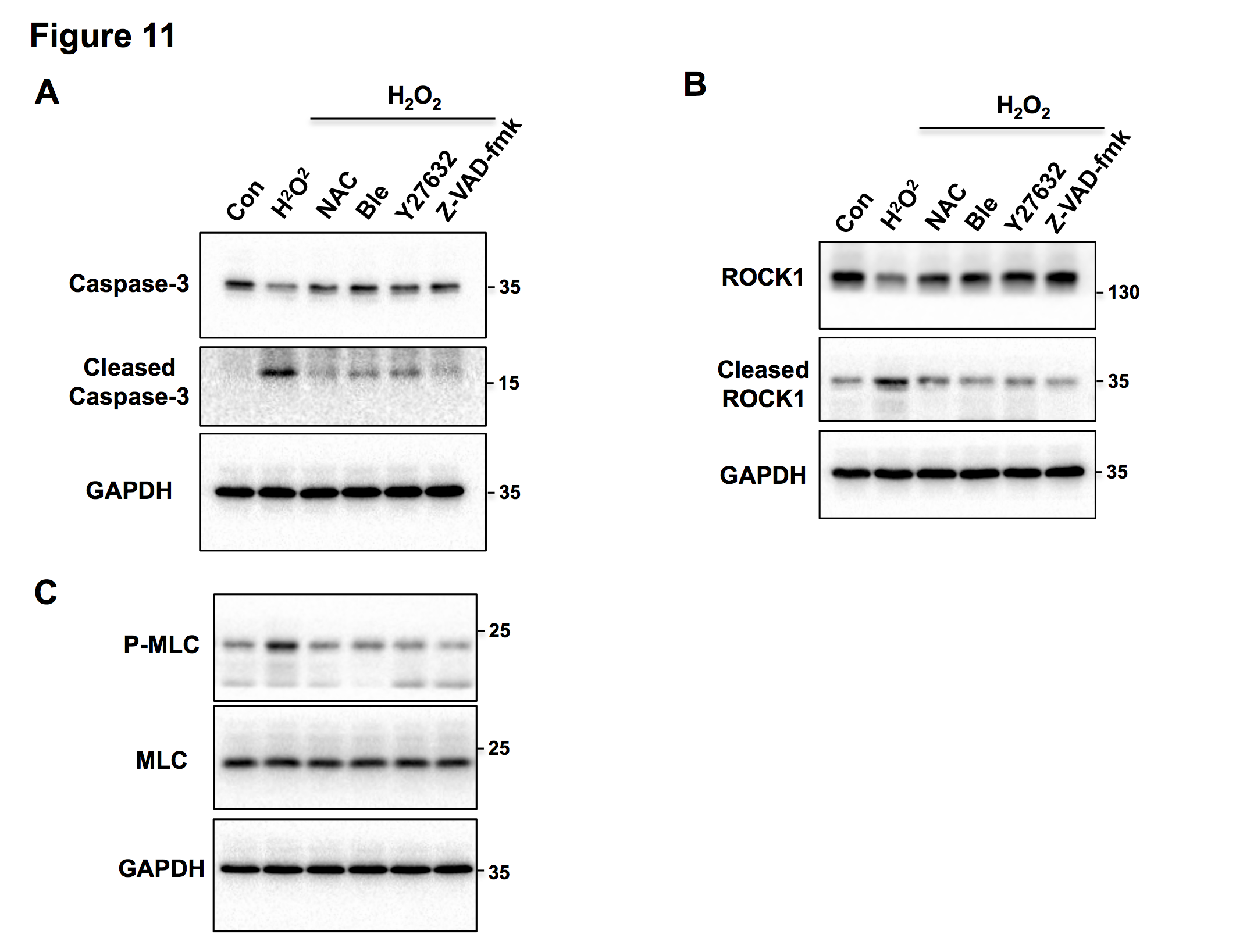
**

**Supplementary Figure S8. Uncropped Western Blot images of figure 11.**


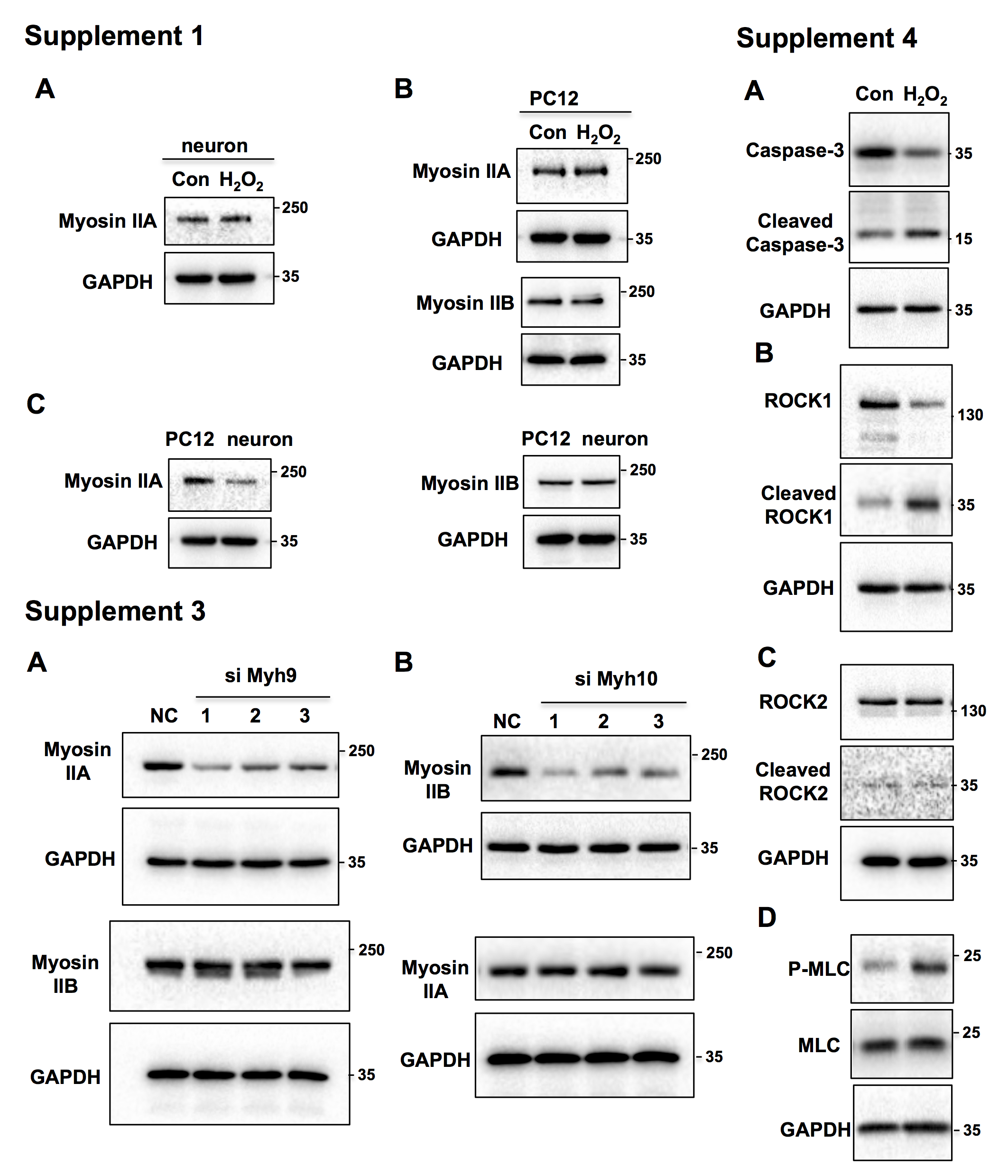


**Supplementary Figure S9. Uncropped Western Blot images of supplementary figure S1, 3 and 4.**
